# Operator differential-algebraic equations with noise arising in fluid dynamics

**DOI:** 10.1007/s00605-016-0931-z

**Published:** 2016-05-24

**Authors:** Robert Altmann, Tijana Levajković, Hermann Mena

**Affiliations:** 1 Institut für Mathematik MA4-5, Technische Universität Berlin, Straße des 17. Juni 136, 10623 Berlin, Germany; 2 Institut für Mathematik, Universität Innsbruck, Technikerstraße 13a, 6020 Innsbruck, Austria; 3 Faculty of Traffic and Transport Engineering, University of Belgrade, Vojvode Stepe 305, 11000 Belgrade, Serbia

**Keywords:** Operator DAE, Noise disturbances, Chaos expansion, Itô-Skorokhod integral, Stochastic convolution, Regularization, 65J10, 60H40, 60H30, 35R60

## Abstract

We study linear semi-explicit stochastic operator differential algebraic equations (DAEs) for which the constraint equation is given in an explicit form. In particular, this includes the Stokes equations arising in fluid dynamics. We combine a white noise polynomial chaos expansion approach to include stochastic perturbations with deterministic regularization techniques. With this, we are able to include Gaussian noise and stochastic convolution terms as perturbations in the differential as well as in the constraint equation. By the application of the polynomial chaos expansion method, we reduce the stochastic operator DAE to an infinite system of deterministic operator DAEs for the stochastic coefficients. Since the obtained system is very sensitive to perturbations in the constraint equation, we analyze a regularized version of the system. This then allows to prove the existence and uniqueness of the solution of the initial stochastic operator DAE in a certain weighted space of stochastic processes.

## Introduction

The governing equations of an incompressible flow of a Newtonian fluid are described by the Navier–Stokes equations [43]. Therein, one searches for the evolution of a velocity field *u* and the pressure *p* to given initial data, a volume force, and boundary conditions. For results on the existence of a (unique) solution, we refer to [20], [42, Ch. 25], and [43, Ch.III].

In this paper, we consider the linear case but allow a more general constraint, namely that the divergence of the velocity does not vanish. Note that this changes the analysis and numerics since the state-of-the-art methods are often tailored for the particular case of a vanishing divergence. An application with non-vanishing divergence is given by the optimal control problem constrained by the Navier–Stokes equations where the cost functional includes the pressure [23].

The Navier–Stokes equations, as well as the corresponding linearized equations, can be formulated as differential-algebraic equations (DAEs) in an abstract setting [3, 4]. These so-called *operator DAEs* correspond to the weak formulation in the framework of partial differential equations (PDEs). As generalization of finite-dimensional DAEs, see [19, 25, 26] for an introduction, also here considered constrained PDEs suffer from instabilities and ill-posedness. This is the reason why the stable approximation of the pressure (which is nothing else than a Lagrange multiplier to enforce the incompressibility) is a great challenge.

One solution strategy is to perform a regularization which corresponds to an *index reduction* in the finite-dimensional setting. With this, the issue of instabilities with respect to perturbations is removed. In the case of fluid dynamics, this has been shown in [4].

In this paper, we study the stochastic version of operator DAEs considered in the framework of white noise analysis and chaos expansions of generalized stochastic processes [18, 21, 39]. More precisely, we consider semi-explicit operator DAEs with perturbations of stochastic type. We combine the polynomial chaos expansion approach from the white noise theory with the deterministic theory of operator DAEs. Particularly, in the fluid flow case, we deal with the stochastic equations of the form

$$\begin{aligned} \begin{array}{cccccccccc} \dot{u}(t) &{} - &{} \Delta u(t) &{} +&{} \nabla p(t) &{} = &{} \mathcal {F}(t) &{}+&{} \text {``noise''}, \\ &{} &{} {{\mathrm{div}}}u(t) &{} &{} &{} = &{} \mathcal {G}(t) &{}+&{} \text {``noise''} \end{array} \end{aligned}$$

with an initial value for *u*(0). In order to preserve the mean dynamics, we deal with stochastic perturbations of zero mean. This implies that the expected value of the stochastic solution equals the solution of the corresponding deterministic operator DAE. For the “noise” processes we consider either a general Gaussian white noise process or perturbations which can be expressed in the form of a stochastic convolution.

Within this paper, we consider the Gaussian white noise space $(\Omega , \mathcal F, \mu )$ with the Gaussian probability measure $\mu$ to be the underlying probability space. Instead, the same analysis can be provided also on Poissonian white noise space $(\Omega , \mathcal F, \nu )$, with the Poissonian probability measure $\nu$, on fractional Gaussian white noise space $(\Omega , \mathcal F, \mu _{H})$, or on fractional Poissonian white noise space $(\Omega , \mathcal F, \nu _H)$, for $H\in (0,1)$. This follows from the existence of unitary mappings between Gaussian and Poissonian white noise spaces, and between Gaussian and fractional Gaussian white noise spaces [27].

With the application of the polynomial chaos expansion method, also known as the *propagator method*, the problem of solving the initial stochastic equations is reduced to the problem of solving an infinite triangular systems of deterministic operator DAEs, which can be solved recursively. Summing up all coefficients of the expansion and proving convergence in an appropriate space of stochastic processes, one obtains the stochastic solution of the initial problem.

The chaos expansion methodology is a very useful technique for solving many types of stochastic differential equations, linear and nonlinear, see e.g. [6, 18, 29, 30, 32–34, 40, 46]. The main statistical properties of the solution, its mean, variance, and higher moments, can be calculated from the formulas involving only the coefficients of the chaos expansion representation [16, 36].

The proposed method allows to apply regularization techniques from the theory of deterministic operator DAEs to the related stochastic system. Applications arise in fluid dynamics, but are not only restricted to this case. The same procedure can be used to regularize other classes of equations that fulfill our setting. A specific example with the operators of the Malliavin calculus is described in Sect. 5. For this reason, in the present paper, we develop a general abstract setting based on white noise analysis and chaos expansions. Numerical experiments with truncated chaos expansions, i.e., stochastic Galerkin methods, are not included in this paper. However, once we regularize each system, it becomes numerically well-posed [3] and then the stochastic equation is well-posed as well.

The paper is organized as follows. In Sect. 2 we introduce the concept of (deterministic) operator DAEs with special emphasis on applications in fluid dynamics. Considering perturbation results for such systems, we detect the necessity of a regularization in order to allow stochastic perturbations. The stochastic setting for the chaos expansion is then given in Sect. 3. Furthermore, we discuss stochastic noise terms in the differential as well as in the constraint equation and the systems which result from the chaos expansions, Theorems 6, 8 and 9. The extension to more general cases is then subject of Sect. 4. Therein, we consider more general operators and stochastic convolution terms. We also provide proofs of the convergence of the obtained solutions in appropriate spaces of generalized stochastic processes, Theorem 11. In Sect. 5 we consider shortly a specific example of DEAs that involve stochastic operators arising in Malliavin calculus. The proof of existence of a unique solution in a space of generalized stochastic processes is given in Theorem 13. Finally, we discuss extensions of our results to specific types of nonlinear equations.

## Operator DAEs

In this section we introduce the concept of operator DAEs, analyze the influence of perturbations, provide regularization of operator DAEs, and state stability results.

### Abstract setting

First we consider operator DAEs (also called PDAEs) which equal constrained PDEs in the weak setting or DAEs in an abstract framework [3, 15]. Thus, we work with generalized derivatives in time and space. In particular, we consider semi-explicit operator DAEs for which the constraint equation is explicitly stated.

We consider real, separable, and reflexive Banach spaces $\mathcal {V}$ and $\mathcal {Q}$ and a real Hilbert space $\mathcal {H}$. Furthermore, we assume that we have a Gel’fand triple of the form

$$\begin{aligned} \mathcal {V} \subseteq \mathcal {H} \subseteq \mathcal {V} ^* , \end{aligned}$$

which means that $\mathcal {V}$ is continuously and densely embedded in $\mathcal {H}$ [47, Ch. 23]. As a consequence, well-known embedding theorems yield the continuous embedding

$$\begin{aligned} \big \{ v\in L^2(T;\mathcal {V} ) :\ \dot{v}\in L^2(T;\mathcal {V} ^*) \big \} \hookrightarrow C(T; \mathcal {H} ). \end{aligned}$$

Note that $L^2(T;\mathcal {V} )$ denotes the Bochner space of abstract functions on a time interval *T* with values in $\mathcal {V}$, see [14, Ch. 7.1] for an introduction. The corresponding norm of $L^2(T;\mathcal {V} )$, which we denote by $\Vert \cdot \Vert _{L^2(\mathcal {V} )}$, is given by

$$\begin{aligned} \Vert u\Vert _{L^2(\mathcal {V} )}^2 := \Vert u\Vert _{L^2(T;\mathcal {V} )}^2 := \int _T \Vert u(t)\Vert _{\mathcal {V} }^2 \ \text {d}t . \end{aligned}$$

The (deterministic) problem of interest has the form

1a $$\begin{aligned} \dot{u}(t) + K u(t) +\ B^* \lambda (t)&= F(t)\quad \text {in }\mathcal {V} ^* , \end{aligned}$$

1b $$\begin{aligned} B u(t)&= G(t)\quad \text {in }\mathcal {Q} ^* , \end{aligned}$$

with (consistent) initial condition $u(0) = u^0 \in \mathcal {H}$. The need of consistent initial values is one characteristic of DAEs in the finite dimensional setting [10, 25]. The condition in the infinite-dimensional case is discussed in Remark 1 below.

Furthermore, we need the operators and right-hand sides of (1 to satisfy the following assumptions.

#### Assumption 1

1. The right-hand sides of (1 satisfy
  $$\begin{aligned} F \in L^2(T;\mathcal {V} ^*) \quad \text {and} \quad G \in H^1(T;\mathcal {Q} ^*) \hookrightarrow C(T; \mathcal {Q} ^*). \end{aligned}$$
2. The constraint operator $B:\mathcal {V} \rightarrow \mathcal {Q} ^*$ is linear and there exists a right-inverse which is denoted by $B^-$.
3. Operator $K:\mathcal {V} \rightarrow \mathcal {V} ^*$ is linear, positive on the kernel of *B*, and continuous.

Note that the involved operators $B:\mathcal {V} \rightarrow \mathcal {Q} ^*$ and $K:\mathcal {V} \rightarrow \mathcal {V} ^*$ can be extended to Nemytskii mappings of the form $B:L^2(T;\mathcal {V} ) \rightarrow L^2(T;\mathcal {Q} ^*)$ and $K:L^2(T;\mathcal {V} ) \rightarrow L^2(T;\mathcal {V} ^*)$, see [41, Ch. 1.3]. From here onwards, we restrict ourselves to the linear case.

As search space for the solution $(u, \lambda )$ we consider

$$\begin{aligned} u \in L^2(T;\mathcal {V} ) \ \text {with}\ \dot{u} \in L^2(T;\mathcal {V} ^*) \quad \text {and}\quad \lambda \in L^2(T;\mathcal {Q} ). \end{aligned}$$

Note that the actual meaning of equation (1a) is that for all test functions $v\in \mathcal {V}$ and $\Phi \in C^\infty (T)$ it holds that

$$\begin{aligned} \int _T \big \langle \dot{u}(t) + K u(t) + B^* \lambda (t), v \big \rangle \, \Phi (t) \ \text {d}t = \int _T \big \langle F(t), v \big \rangle \, \Phi (t) \ \text {d}t . \end{aligned}$$

#### Remark 1

(Consistent initial values) DAEs require consistent initial data because of the given constraints which also apply to the initial condition. This remains valid for the operator case. However, since we allow $u^0 \in \mathcal {H}$, the constraint operator *B* is not applicable to $u^0$. In this case, the condition has the form

$$\begin{aligned} u^0 = u^0_B + B^- G(0) \end{aligned}$$

where $u^0_B$ is an arbitrary element from the closure of the kernel of *B* in $\mathcal {H}$ [4, 15]. If $u^0 \in \mathcal {V}$ is given, then we get the same decomposition but with $u^0_B \in {{\mathrm{Ker}}}B$.

In the following, we write $a\lesssim b$ meaning that there exists a positive constant *c* such that $a\le cb$. We show that the solution is bounded in terms of the initial data, the right-hand sides, and their derivative, cf. [3, Sect. 6.1.3].

#### Theorem 1

(Stability estimate) Given Assumption 1 and consistent initial data $u^0= u^0_B + B^- G(0)\in \mathcal {H}$, the solution of the operator DAE (1 satisfies the estimate

2 $$\begin{aligned} \Vert u\Vert _{L^2(\mathcal {V} )}^2 \lesssim \left\| u^0_B\right\| _\mathcal {H} ^2 + \Vert F\Vert ^2_{L^2(\mathcal {V} ^*)} + \Vert G\Vert ^2_{H^1(\mathcal {Q} ^*)}. \end{aligned}$$

#### Proof

We consider a splitting of the space $\mathcal {V} =\mathcal {V}_{B}\oplus \mathcal {V}^\text {c}$ which we will also use later within the regularization in Sect. 2.3. Therein, $\mathcal {V}_{B}$ denotes the kernel of the operator *B* and $\mathcal {V}^\text {c}$ is any complementary space. This gives a unique decomposition $u=u_1+u_2$ where $u_1$, $u_2$ take values in $\mathcal {V}_{B}$, $\mathcal {V}^\text {c}$, respectively. Thus, we have $Bu = B u_2 = G$ and therefore $u_2 = B^- G$. The assumption on *G* implies $u_2 \in H^1(T;\mathcal {V} )$ and

$$\begin{aligned} \Vert u_2 \Vert _{L^2(\mathcal {V} )} \lesssim \Vert G \Vert _{L^2(\mathcal {Q} ^*)}, \qquad \Vert \dot{u}_2 \Vert _{L^2(\mathcal {V} )} \lesssim \Vert \dot{G} \Vert _{L^2(\mathcal {Q} ^*)}. \end{aligned}$$

It remains to find a bound of $u_1$. For this, we insert $u_1$ in (1a) as test function which leads to

$$\begin{aligned} \frac{1}{2}\frac{\text {d}}{\text {d}t} \Vert u_1\Vert _\mathcal {H} ^2 + \Vert u_1\Vert _\mathcal {V} ^2&\lesssim \langle \dot{u}_1, u_1\rangle + \langle \mathcal {K} u_1, u_1 \rangle \\&= \langle F, u_1\rangle - \langle \dot{u}_2, u_1\rangle - \langle \mathcal {K} u_2, u_1 \rangle \\&\lesssim \Vert F\Vert _{\mathcal {V} ^*} \Vert u_1\Vert _\mathcal {V} + \Vert \dot{G}\Vert _{\mathcal {Q} ^*} \Vert u_1\Vert _\mathcal {V} + \Vert G\Vert _{\mathcal {Q} ^*} \Vert u_1 \Vert _\mathcal {V} . \end{aligned}$$

Note that the Lagrange multiplier $\lambda$ vanishes, since the test function is element of $\mathcal {V}_{B}$. Thus, by the Young’s inequality we obtain

$$\begin{aligned} \frac{\text {d}}{\text {d}t} \Vert u_1\Vert _\mathcal {H} ^2 + \Vert u_1\Vert _\mathcal {V} ^2 \lesssim \Vert F \Vert _{\mathcal {V} ^*}^2 + \Vert G \Vert _{\mathcal {Q} }^2 + \Vert \dot{G}\Vert ^2_{\mathcal {Q} ^*}. \end{aligned}$$

An integration of this estimate over the given time interval $T=[0, t_\text {end}]$ finally leads to

$$\begin{aligned} \Vert u_1(t_\text {end})\Vert _\mathcal {H} ^2 + \Vert u_1\Vert _{L^2(\mathcal {V} )}^2 \lesssim \Vert u_1(0)\Vert _\mathcal {H} ^2 + \Vert F \Vert _{L^2(\mathcal {V} ^*)}^2 + \Vert G \Vert _{L^2(\mathcal {Q} )}^2 + \Vert \dot{G}\Vert ^2_{L^2(\mathcal {Q} ^*)}. \end{aligned}$$

This completes the proof, since $u_1(0)=u^0_B$. $\square$

#### Remark 2

Throughout the paper, we concentrate on results for the variable *u* which corresponds to the velocity in terms of fluid flow applications. Similar results for the Lagrange multiplier $\lambda$ (respectively the pressure) are valid but require stronger regularity assumptions on *F* and $u^0$. For a detailed stability analysis of the Lagrange multiplier, we refer to [50, Ch. 3.1.2]. Note that Assumption 1 is not sufficient to prove $\lambda \in L^2(T;\mathcal {Q} )$.

Since this paper focuses on fluid flows, we show that the linear Stokes equations fit into the given framework. Note that also the Navier–Stokes equations may be considered in the given setting if we allow the operator *K* in (1 to be nonlinear. However, we exclude the nonlinear case in this paper.

#### Example 1

(Stokes equations) The linear Stokes equations provide a leading-order simplification of the Navier–Stokes equations and describe the incompressible flow of a Newtonian fluid in a bounded domain *D*, cf. [43]. We consider homogeneous Dirichlet boundary conditions and set

$$\begin{aligned} \mathcal {V} = [H^1_0(D)]^d,\qquad \mathcal {H} = [L^2(D)]^d, \qquad \mathcal {Q} = L^2(D)/\mathbb {R}. \end{aligned}$$

Furthermore, we define $G \equiv 0$, $B = {{\mathrm{div}}}$ with dual operator $B^* = - \nabla$, and *K* which equals the weak form of the Laplace operator, i.e.,

$$\begin{aligned} \langle K u, v\rangle := \int _D \nabla u \cdot \nabla v\ \text {d}x . \end{aligned}$$

The solution *u* describes the velocity of the fluid whereas $\lambda$ measures the pressure. The operator equations (1 then equal the weak formulation of the Stokes equations

$$\begin{aligned} \dot{u} - \Delta u + \nabla \lambda = f, \qquad \nabla \cdot u = 0, \qquad u(0) = u^0. \end{aligned}$$

For the Stokes equations with stochastic noise, we refer to Example 7 below.

#### Example 2

(Linearized Navier–Stokes equations) With a simple modification of the operator *K*, the framework given in Example 1 includes any linearization of the Navier–Stokes equations such as the Oseen equations.

Given the characteristic velocity $u_\infty$, the Oseen equations include the operator

$$\begin{aligned} \langle K u, v\rangle := \int _D (u_\infty \cdot \nabla )u v + \nu \nabla u \cdot \nabla v\ \text {d}x \end{aligned}$$

or even

$$\begin{aligned} \langle K u, v\rangle := \int _D (u_\infty \cdot \nabla )u v + (u\cdot \nabla )u_\infty v + \nu \nabla u \cdot \nabla v\ \text {d}x . \end{aligned}$$

Note that *u* describes the ’disturbance velocity’, i.e., the variation around $u_\infty$.

Although we focus here on applications in fluid dynamics, we emphasize that the given framework is not restricted to this class. Further examples are given by PDEs with boundary control [11] (with *B* being the trace operator) as well as applications in elastodynamics which leads to second-order systems of similar structure [2].

### Influence of perturbations

DAEs are known for its high sensitivity to perturbations. The reason for this is that derivatives of the right-hand sides appear in the solution. In particular, this implies that a certain smoothness of the right-hand sides is necessary for the existence of solutions. Furthermore, the numerical approximation is much harder than for ODEs, since small perturbations - such as round-off errors or errors within iterative methods - may have a large influence [38].

The resulting level of difficulty in the numerical approximation of DAEs is measured by the so-called *index*. There exist several index concepts [35] and we use here the *differentiation index*, see [10, Def. 2.2.2] for a precise definition. A comparable index concept for operator DAEs which may be used to classify systems of the form (1 does not exist. Thus, in order to obtain information about stability issues it is advisable to analyse the influence of perturbations. Furthermore, a spatial discretization of system (1 by finite elements (under some basic assumptions) leads to a DAE of index 2. Note that the understanding of the index is not crucial for the further reading of this paper. However, we comment on the index from time to time for additional insight.

We consider system (1 with additional perturbations $\delta \in L^2(T;\mathcal {V} ^*)$ and $\theta \in H^1(T;\mathcal {Q} ^*)$. The perturbed solution $(\hat{u}, \hat{\lambda })$ then satisfies the system

$$\begin{aligned} \dot{\hat{u}}\ +\ K \hat{u} \ +\ B^* \hat{\lambda }&=\ F+\delta \quad \text {in }\mathcal {V} ^*,\\ B\hat{u}&=\ G+\theta \quad \text {in }\mathcal {Q} ^*. \end{aligned}$$

Let $e_1$ denote the difference of *u* and $\hat{u}$ projected to the kernel of the constraint operator *B*. Accordingly, we denote the projected initial error by $e_{1,0}$. In [5] it is shown that with the given assumptions on the operators *K* and *B* of Assumption 1, we have

3 $$\begin{aligned} \Vert e_1\Vert ^2_{C(T;\mathcal {H} )} + \Vert e_1\Vert ^2_{L^2(\mathcal {V} )} \lesssim \Vert e_{1,0} \Vert _\mathcal {H} ^2 + \Vert \delta \Vert _{L^2(\mathcal {V} ^*)}^2 + \Vert \theta \Vert _{L^2(\mathcal {Q} ^*)}^2 + \Vert \dot{\theta }\Vert _{L^2(\mathcal {Q} ^*)}^2. \end{aligned}$$

This estimate shows that the error depends on the derivative of the perturbation $\theta$. Note that this is crucial if we consider stochastic perturbations in Sect. 3 where we apply the chaos expansion method to reduce the given problem to an infinite number of deterministic systems. Similar to index reduction procedures for DAEs, cf. [10, 25], the operator DAE can be regularized in view of an improved behaviour with respect to perturbations.

### Regularization of operator DAEs

In this subsection, we introduce an operator DAE which is equivalent to (1, but where the solution of the perturbed system does not depend on derivatives of the perturbations. Furthermore, a semi-discretization in space of the regularized system directly leads to a DAE of index 1 and thus, is better suited for numerical integration [25].

In the case of the Stokes equations, the right-hand side *G* vanishes since we search for divergence-free velocities. In this case, the constrained system is often reduced to the kernel of the constraint operator *B* which leads to an operator ODE, i.e., a time-dependent PDE. However, with the stochastic noise term in the constraint, we cannot ignore the inhomogeneity anymore. In addition, the inclusion of *G* enlarges the class of possible applications. Thus, we propose to apply a regularization of the operator DAE.

For the regularization we follow the procedure introduced first in [2] for second-order systems. The idea is to add the derivative of the constraint, the so-called *hidden constraint*, to the system. In order to balance the number of equations and variables, we add a so-called *dummy variable* $v_2$ to the system. The assumptions are as before, but we split the space $\mathcal {V}$ into $\mathcal {V} =\mathcal {V}_{B}\oplus \mathcal {V}^\text {c}$ were

$$\begin{aligned} \mathcal {V}_{B}:= {{\mathrm{Ker}}}B \end{aligned}$$

and $\mathcal {V}^\text {c}$ is any complementary space on which *B* is invertible, i.e., there exists a right-inverse of *B*, namely $B^-:\mathcal {Q} ^* \rightarrow \mathcal {V}^\text {c}$ with $B B^- q=q$ for all $q\in \mathcal {Q} ^*$. In the example of the Stokes equations, cf. Example 1, $\mathcal {V}_{B}$ is the space of divergence-free functions which build a proper subspace of $\mathcal {V}$ and $\mathcal {V}^\text {c}$ equals its orthogonal complement in $\mathcal {V}$. We then search for a solution $(u_1, u_2, v_2, \lambda )$ where $u_1$ takes values in $\mathcal {V}_{B}$ and $u_2$, $v_2$ in the complement $\mathcal {V}^\text {c}$. The extended (but equivalent) system then reads

4a $$\begin{aligned} \dot{u}_1(t)\ + v_2(t) + K \big (u_1(t)+u_2(t)\big )\ +\ B^* \lambda (t)&= F(t)\quad \text {in }\mathcal {V} ^*, \end{aligned}$$

4b $$\begin{aligned} B u_2(t)&= G(t)\quad \text {in }\mathcal {Q} ^*, \end{aligned}$$

4c $$\begin{aligned} B v_2(t)&= \dot{G}(t)\quad \text {in }\mathcal {Q} ^* \end{aligned}$$

with initial condition

4d $$\begin{aligned} u_1(0)&= u^0_B - B^-G(0) \in \mathcal {H} . \end{aligned}$$

Recall that $u^0_B$ is an element of the closure of $\mathcal {V}_{B}$ in $\mathcal {H}$, cf. Remark 1. The connection of system (1 and (4 is given by $u=u_1+u_2$ and $v_2 = \dot{u}_2$. Note, however, that in system (4 $u_2$ is not differentiated anymore and corresponds to an algebraic variable in the finite-dimensional case. For the regularized formulation (4 we obtain the following stability result.

#### Theorem 2

(Influence of perturbations) Let Assumption 1 be satisfied and consider perturbations $\delta \in L^2(T;\mathcal {V} ^*)$ and $\theta , \xi \in L^2(T;\mathcal {Q} ^*)$ of the right-hand sides of (4 with the corresponding perturbed solution $(\hat{u}_1, \hat{u}_2, \hat{v}_2, \hat{\lambda })$. Then, the error in $u_1$, namely $e_1 = \hat{u}_1 - u_1$, satisfies the estimate

5 $$\begin{aligned} \Vert e_1\Vert ^2_{C(T;\mathcal {H} )} + \Vert e_1\Vert ^2_{L^2(\mathcal {V} )} \lesssim \Vert e_{1,0} \Vert _\mathcal {H} ^2 + \Vert \delta \Vert _{L^2(\mathcal {V} ^*)}^2 + \Vert \theta \Vert _{L^2(\mathcal {Q} ^*)}^2 + \Vert \xi \Vert _{L^2(\mathcal {Q} ^*)}^2 . \end{aligned}$$

#### Proof

We introduce the remaining errors $e_2 := \hat{u}_2 - u_2$, $e_v := \hat{v}_2 - v_2$, and $e_\lambda := \hat{\lambda }- \lambda$. The difference of the original and the perturbed problem then yields an operator DAE for $e_1$, $e_2$, $e_v$, and $e_\lambda$ of the form (4, namely

$$\begin{aligned} \dot{e}_1(t)\ + e_v(t) + K \big (e_1(t)+e_2(t)\big )\ +\ B^* e_\lambda (t)&= \delta (t)\quad \text {in }\mathcal {V} ^*, \\ B e_2(t)&= \theta (t)\quad \text {in }\mathcal {Q} ^*, \\ B e_v(t)&= \xi (t)\quad \text {in }\mathcal {Q} ^* \end{aligned}$$

with initial condition $e_1(0) = e_{1,0}$. From this point on, we follow the arguments of the proof of Theorem 1, using

$$\begin{aligned} \Vert e_2 \Vert _{L^2(\mathcal {V} )} \lesssim \Vert \theta \Vert _{L^2(\mathcal {Q} ^*)}, \qquad \Vert e_v \Vert _{L^2(\mathcal {V} )} \lesssim \Vert \xi \Vert _{L^2(\mathcal {Q} ^*)} \end{aligned}$$

instead of the estimates of $u_2$ and $\dot{u}_2$ therein. Thus, we obtain the estimate

$$\begin{aligned} \Vert e_1(t)\Vert _\mathcal {H} ^2 + \Vert e_1\Vert _{L^2(\mathcal {V} )}^2 \lesssim \Vert e_1(0)\Vert _\mathcal {H} ^2 + \Vert \delta \Vert _{L^2(\mathcal {V} ^*)}^2 + \Vert \theta \Vert _{L^2(\mathcal {Q} ^*)}^2 + \Vert \xi \Vert ^2_{L^2(\mathcal {Q} ^*)} \end{aligned}$$

for all $t \in T$. Thus, maximizing over *t* and using the initial condition, we obtain the stated assertion. $\square$

Note that, in contrast to the original formulation, estimate (5) does not depend on derivatives of the perturbations. This is crucial when we consider stochastic perturbations.

## Inclusion of stochastic perturbations

In this section, we consider the operator DAE (1 with additional stochastic perturbation terms, also called noise terms. Clearly, we perturb the deterministic system with zero mean disturbances. First, we consider the noise, only in the differential equation, i.e., we study

6a $$\begin{aligned} {\dot{u}}(t) + {\mathcal {K} } u(t) + {{\mathcal {B} }^{*}} {\lambda } (t)&= {\mathcal {F} (t)} + \text {``noise''}, \end{aligned}$$

6b $$\begin{aligned} \mathcal {B} u(t)&=\ \mathcal {G} (t). \end{aligned}$$

Afterwards, we also add a noise term in the constraint equation,

7a $$\begin{aligned} {\dot{u}}(t) + {\mathcal {K} } u(t) + {\mathcal {B} }^{*} {\lambda } (t)&= {\mathcal {F} (t)} + \text {``noise''}, \end{aligned}$$

7b $$\begin{aligned} {\mathcal {B} } u(t)&= {\mathcal {G} (t)} + \text {``noise''}. \end{aligned}$$

As discussed in Sect. 2.2, perturbations in the second equation, i.e., in the constraint equation, lead to instabilities. Thus, we also consider the regularized operator equations (4 with stochastic perturbations. In any case, we assume a consistent initial condition of the form $u(0) =u^0$. Note that with the inclusion of stochastic perturbations, we also allow the initial data $u^0$ to be random.

From the modeling point of view, noise may enter the physical system either as temporal fluctuations of internal degrees of freedom or as random variations of some external control parameters; internal randomness often reflects itself in additive noise terms, while external fluctuations gives rise to multiplicative noise terms. Moreover, the additive noises may appear in various forms, ranging from the space time white noise to colored noises generated by some infinite dimensional Brownian motion with a prescribed covariance operator [13].

### Preliminaries

In this section, we recall some basic facts and notions of the white noise theory, random variables, stochastic processes, and operators. Then we apply the chaos expansion method in order to solve stated problems.

#### White noise space

We consider stochastic DAEs in the white noise framework. For this, the spaces of stochastic test and generalized functions are built by use of series decompositions via orthogonal functions as a basis with certain weight sequences. The classical Hida approach [21] suggests to start with a Gel’fand triple

$$\begin{aligned} \mathcal E \, \subseteq \, L^2(\mathbb R) \, \subseteq \, \mathcal E ' , \end{aligned}$$

with continuous inclusions, formed by a nuclear space $\mathcal E$ and its dual $\mathcal E '$. As basic probability space we set $\Omega = \mathcal E '$ endowed with the Borel sigma algebra of the weak topology and an appropriate probability measure, see [21, 22]. Without loss of generality, in this paper we assume that the underlying probability space is the Gaussian white noise probability space $(S ' (\mathbb R), \mathcal B, \mu )$. Therefore, we take $\mathcal E$ and $\mathcal E '$ to be the *Schwartz spaces* of rapidly decreasing test functions $S(\mathbb R)$ and tempered distributions $S ' (\mathbb R)$, respectively, and $\mathcal B$ the Borel sigma algebra generated by the weak topology on $S ' (\mathbb R)$. By the Bochner-Minlos theorem, there exists a unique measure $\mu$ on $( S ' (\mathbb R), \mathcal B)$ such that for each $\phi \in S(\mathbb R)$ the relation

$$\begin{aligned} \int _{S'(\mathbb R)} \, e^{\langle \omega , \phi \rangle } \, d \mu (\omega ) \, = \, e^{-\frac{1}{2} \, \Vert \phi \Vert ^2_{L^2(\mathbb R)}} \end{aligned}$$

holds, where $\langle \omega , \phi \rangle$ denotes the action of a tempered distribution $\omega \in S'(\mathbb R)$ on a test function $\phi \in S(\mathbb R)$. We denote by $L^2(\Omega , \mu )$, or in short $L^2(\Omega )$, the space of square integrable random variables $L^2(\Omega ) = L^2(\Omega , \mathcal B, \mu )$. It is the Hilbert space of random variables which have finite second moments. Here, the scalar product is $(F,G)_{L^2(\Omega )} = \mathbb E_\mu (F \cdot G)$, where $\mathbb E_\mu$ denotes the expectation with respect to the measure $\mu$. In the sequel, we omit $\mu$ and simply write $\mathbb E$.

In the case of a Gaussian measure, the orthogonal polynomial basis of $L^2(\Omega )$ can be represented as a family of orthogonal *Fourier-Hermite polynomials* defined by use of the Hermite functions and the Hermite polynomials. We denote by $\{h_{n}(x)\}_{n\in \mathbb {N}_0}$ the family of *Hermite polynomials* and $\{\xi _{n}(x)\}_{n\in \mathbb {N}}$ the family of *Hermite functions*, where

$$\begin{aligned} h_{n}(x)&=(-1)^n \, e^{\frac{x^2}{2}} \, \, \frac{d^n}{dx^n}\left( e^{-\frac{x^2}{2}}\right) , \quad \qquad \quad n\in \mathbb N_0 ,\\ \xi _n (x)&= \frac{1}{\root 4 \of {\pi }\sqrt{(n-1)!}} \,\, e^{-\frac{x^2}{2}} \,\, h_{n-1}\left( \sqrt{2}x\right) ,\quad n\in \mathbb N , \end{aligned}$$

for $x\in \mathbb R$. The family of Hermite polynomials forms an orthogonal basis of the space $L^2(\mathbb R)$ with respect to the Gaussian measure $d\mu =\frac{1}{\sqrt{2\pi }}e^{-\frac{x^2}{2}}dx$, while the family of Hermite functions forms a complete orthonormal system in $L^2(\mathbb {R})$ with respect to the Lebesque measure. We follow the characterization of the Schwartz spaces in terms of the Hermite basis [17]. Clearly, the Schwartz space of rapidly decreasing functions can be constructed as the projective limit of the family of spaces

$$\begin{aligned} S_l (\mathbb R)= \left\{ f (t) = \sum _{k\in \mathbb N} a_k \, \xi _k(t) \in L^2(\mathbb R):\ \Vert f\Vert ^2_{l} = \sum _{k\in \mathbb N} \, a_k^2 \, (2k)^l < \infty \right\} , \, \, l\in \mathbb N_0. \end{aligned}$$

The Schwartz space of tempered distributions is isomorphic to the inductive limit of the family of spaces

$$\begin{aligned} S_{- l}(\mathbb R)=\left\{ F (t) = \sum _{k\in \mathbb N} b_k \, \xi _k(t):\ \Vert F\Vert ^2_{- l} = \sum _{k\in \mathbb N} \, b_k^2 \, (2k)^{-l} < \infty \right\} , \, \, l\in \mathbb N_0. \end{aligned}$$

It holds that $S(\mathbb R) = \bigcap _{l\in \mathbb N_0} S_l(\mathbb R)$ and $S'(\mathbb R) = \bigcup _{l\in \mathbb N_0} \, S_{-l}(\mathbb R)$. The action of a generalized function $F = \sum _{k\in \mathbb N} b_k \, \xi _k \in S'(\mathbb R)$ on a test function $f = \sum _{k\in \mathbb N} a_k \, \xi _k \in S(\mathbb R)$ is given by $\langle F, f \rangle = \sum _{k\in \mathbb N} a_k \, b_k$.

#### Spaces of random variables

Let $\mathcal {I}=(\mathbb {N}_{0}^{\mathbb {N}})_{c}$ be the set of sequences of non-negative integers which have only finitely many nonzero components $\alpha =(\alpha _1,\alpha _2,\ldots ,\alpha _m,0,0,\ldots )$, $\alpha _i\in \mathbb {N}_0$, $i=1, 2,\dots , m$, $m\in \mathbb {N}$. The *k*-th unit vector $\varepsilon ^{(k)}=(0,\ldots ,0,1,0,\ldots ), \, k\in \mathbb {N}$, is the sequence of zeros with the entry 1 as the *k*-th component and $\mathbf{0}$ is the multi-index with only zero components. The length of a multi-index $\alpha \in \mathcal {I}$ is defined as $|\alpha |=\sum _{k=1}^\infty \alpha _k$. We say $\alpha \ge \beta$ if $\alpha _k \ge \beta _k$ for all $k\in \mathbb N$ and thus $\alpha -\beta = (\alpha _1-\beta _1, \alpha _2-\beta _2,\ldots )$. For $\alpha < \beta$ the difference $\alpha -\beta$ is not defined. Particularly, for $\alpha _k >0$ we have $\alpha -\varepsilon ^{(k)} = (\alpha _1,\ldots , \alpha _{k-1}, \alpha _k - 1, \alpha _{k+1},\ldots , \alpha _m, 0,\ldots )$, $k\in \mathbb N$. We denote $(2\mathbb {N})^\alpha =\prod _{k=1}^\infty (2k)^{\alpha _k}$.

##### Theorem 3

([49]) It holds that $\sum \nolimits _{\alpha \in \mathcal {I}}(2\mathbb {N})^{-p\alpha }<\infty$ if and only if $p>1$.

The proof can be foud in the paper of Zhang [49], also in [22, Prop. 2.3.3].

We define by

$$\begin{aligned} H_{\alpha }(\omega )=\prod _{k=1}^\infty h_{\alpha _k}\left( \left\langle \omega , \xi _k\right\rangle \right) ,\quad \alpha \in \mathcal I, \end{aligned}$$

the *Fourier-Hermite* orthogonal polynomial basis of $L^2(\Omega )$ such that $\Vert H_\alpha \Vert ^2_{L^2(\Omega )} = \mathbb E (H_\alpha )^2 = \alpha !$. In particular, $H_{\mathbf{0}} (\omega ) = H_{(0,0,\ldots )} (\omega )=1$, and for the *k*-th unit vector $H_{\varepsilon ^{(k)}}(\omega )=h_1(\langle \omega ,\xi _{k}\rangle ) = \langle \omega ,\xi _{k}\rangle$, $k\in \mathbb {N}$.

##### Theorem 4

([22]) (Wiener-Itô chaos expansion theorem) Each random variable $f\in L^2(\Omega )$ has a unique representation of the form

$$\begin{aligned} f(\omega ) = \sum _{\alpha \in \mathcal {I}} \, a_{\alpha } \, H_{\alpha }(\omega ), \quad a_\alpha \in \mathbb {R}, \, \, \omega \in \Omega \end{aligned}$$

such that it holds

$$\begin{aligned} \Vert f\Vert ^2_{L^2(\Omega )}=\sum _{\alpha \in \mathcal {I}} \, a_{\alpha }^2 \, \alpha ! \, <\infty . \end{aligned}$$

The spaces of generalized random variables are stochastic analogues of deterministic generalized functions. They have no point value for $\omega \in \Omega$ but an average value with respect to a test random variable. Following the idea of the construction of $S'(\mathbb R)$ as an inductive limit space over $L^2(\Omega )$ with appropriate weights [48], one can define stochastic generalized random variable spaces over $L^2(\Omega )$ by adding certain weights in the convergence condition of the series expansion. Several spaces of this type, weighted by a sequence $q = (q_\alpha )_{\alpha \in \mathcal I}$, denoted by $(Q)_{-\rho }$, for $\rho \in [0, 1]$ were described in [27]. Thus a Gel’fand triple

$$\begin{aligned} (Q)_{\rho } \, \subseteq \, L^2(\Omega )\, \subseteq \, (Q)_{-\rho } \end{aligned}$$

is obtained, where the inclusions are again continuous. The most common weights and spaces appearing in applications are $q_\alpha =(2\mathbb {N})^{\alpha }$ which correspond to the Kondratiev spaces of stochastic test functions $(S)_{\rho }$ and stochastic generalized functions $(S)_{-\rho }$, for $\rho \in [0,1]$. Exponential weights $q_\alpha =e^{(2\mathbb {N})^{\alpha }}$ are linked with the *exponential growth spaces* of stochastic test functions $\exp (S)_{\rho }$ and stochastic generalized functions $\exp (S)_{-\rho }$ [21, 22, 27, 39, 40]. In this paper, we consider the largest Kondratiev space of stochastic distributions, i.e., $\rho =1$. For definition of the Kondratiev spaces we follow [22].

The space of the *Kondratiev test random variables* $(S)_1$ can be constructed as the projective limit of the family of spaces

$$\begin{aligned} (S)_{1,p}= \left\{ f (\omega ) = \sum _{\alpha \in \mathcal I} a_\alpha H_\alpha (\omega )\in L^2(\Omega ):\Vert f\Vert ^2_{1,p} = \sum \limits _{\alpha \in \mathcal I}a_\alpha ^2(\alpha !)^{2}(2\mathbb {N})^{p\alpha }<\infty \right\} , \end{aligned}$$

$p\in \mathbb N_0$. The space of the *Kondratiev generalized random variables* $(S)_{-1}$ can be constructed as the inductive limit of the family of spaces

$$\begin{aligned} (S)_{-1, -p}= \left\{ F (\omega ) = \sum _{\alpha \in \mathcal I} b_\alpha \, H_\alpha (\omega ):\ \Vert f\Vert ^2_{-1,-p} = \sum \limits _{\alpha \in \mathcal I} b_\alpha ^2 \, (2\mathbb {N})^{-p\alpha }<\infty \right\} , p\in \mathbb N_0. \end{aligned}$$

It holds that $(S)_1 = \bigcap _{p\in \mathbb N_0} (S)_{1,p}$ and $(S)_{-1} = \bigcup _{p\in \mathbb N_0} (S)_{-1,p}$. The action of a generalized random variable $F = \sum _{\alpha \in \mathcal I} b_\alpha \, H_\alpha (\omega ) \in (S)_{-1}$ on a test random variable $f = \sum _{\alpha \in \mathcal I} b_\alpha \, H_\alpha (\omega ) \in (S)_{1}$ is given by $\langle F, f \rangle = \sum _{\alpha \in \mathcal I} \alpha ! \, a_\alpha \, b_\alpha$. It holds that $(S)_1$ is a nuclear space with the Gel’fand triple structure

$$\begin{aligned} (S)_1\subseteq L^2(\Omega )\subseteq (S)_{-1} , \end{aligned}$$

with continuous inclusions. Moreover, for $0\le p \le q$ it holds $(S)_{1, q} \subseteq (S)_{1, p} \subseteq (S)_{1, 0} \subseteq L^2(\Omega ) \subseteq (S)_{-1, 0} \subseteq (S)_{-1, -p} \subseteq (S)_{-1, -q}$. The proof of nuclearity of $(S)_{1}$ can be found in [21] and in [22, Lemma 2.8.2].

The problem of pointwise multiplications of generalized stochastic functions in the white noise analysis is overcome by introducing the *Wick product*, which represents the stochastic convolution. The fundamental theorem of stochastic calculus states the relation of the Wick multiplication to the Itô-Skorokhod integration [22].

Let *L* and *S* be random variables given in their chaos expansion representations $L= \sum _{\alpha \in \mathcal I} \ell _\alpha H_\alpha$ and $S= \sum _{\alpha \in \mathcal I} s_\alpha \, H_\alpha$, $\ell _\alpha , s_\alpha \in \mathbb R$ for all $\alpha \in \mathcal I$. Then, the *Wick product* $L\lozenge S$ is defined by

8 $$\begin{aligned} L\lozenge S \, =\, \sum _{\gamma \in \mathcal I}\left( \sum _{\alpha +\beta =\gamma }\ell _\alpha s_\beta \right) H_\gamma (\omega ). \end{aligned}$$

Note here that the space $L^2(\Omega )$ is not closed under the Wick multiplication.

##### Example 3

Consider the random variable $F= \sum _{k\in \mathbb N} \, \frac{1}{k}\, H_{\varepsilon ^{(k)}}$ and its Wick square $F^{\lozenge \, 2} = F\lozenge F= \sum _{n=1}^\infty \sum _{k=0}^{n-1} \, \frac{1}{n \, (n-k)} \, H_{\varepsilon ^{(n)}}$. Then $F\in L^2(\Omega )$, since $\Vert F\Vert ^2_{L^2(\Omega )} = \sum _{k\in \mathbb N} \, \frac{1}{k^2} < \infty$. In contrast, its Wick square $F^{\lozenge \, 2}$ is not an element of $L^2(\Omega )$, since it holds that

$$\begin{aligned} \sum _{n=1}^\infty \, \left( \sum _{k=0}^{n-1} \, \frac{1}{n \, (n-k)} \right) ^2 \ge \sum _{k=1}^\infty \frac{1}{k\, (k+1)} = \sum _{k=1}^\infty \left( 1- \frac{1}{k+1}\right) = + \infty . \end{aligned}$$

Kondratiev spaces $(S)_1$ and $(S)_{-1}$ are closed under the Wick multiplication. For the proof we refer to [22, Lemma 2.4.4].

#### Stochastic processes

Classical stochastic process can be defined as a family of functions $v:T \times \Omega \rightarrow \mathbb R$ such that for each fixed $t \in T$, $v(t,\cdot )$ is an $\mathbb R$-valued random variable and for each fixed $\omega \in \Omega$, $v(\cdot , \omega )$ is an $\mathbb R$-valued deterministic function, called *trajectory*. Here, following [39], we generalize the definition of a classical stochastic process and define generalized stochastic processes. By replacing the space of trajectories with some space of deterministic generalized functions, or by replacing the space of random variables with some space of generalized random variables, different types of generalized stochastic processes can be obtained. In this manner, we obtain processes generalized with respect to the *t* argument, the $\omega$ argument, or even with respect to both arguments [22, 39].

A very general concept of generalized stochastic processes, based on chaos expansions was introduced in [39] and further developed in [27, 28]. In [22] generalized stochastic processes are defined as measurable mappings $T \rightarrow (S)_{-1}$. Thus, they are defined pointwise with respect to the parameter $t \in T$ and generalized with respect to $\omega \in \Omega$. We define such processes by their chaos expansion representations in terms of an orthogonal polynomial basis.

Let $\tilde{X}$ be a Banach space endowed with the norm $\Vert \cdot \Vert _{\tilde{X}}$ and let $\tilde{X}'$ denote its dual space. If, for example, $\tilde{X}$ is a space of functions on $\mathbb R$ such as $\tilde{X}=C^k(T)$ or $\tilde{X}=L^2(\mathbb R)$, we obtain stochastic processes. The definition of processes where $\tilde{X}$ is not a normed space, but a nuclear space topologized by a family of seminorms, e.g. $\tilde{X}=S(\mathbb R)$ is given in [39].

Let *u* have the formal expansion $u=\sum _{\alpha \in \mathcal I} u_\alpha \otimes H_\alpha$, where $f_\alpha \in X$ and $\alpha \in \mathcal I$. We define the spaces

$$\begin{aligned} \begin{array}{ll} X\otimes (S)_{1,p} &{}=\left\{ f: \Vert f\Vert ^2_{X\otimes (S)_{1,p}} = \sum \limits _{\alpha \in \mathcal I}\alpha !^2\Vert f_\alpha \Vert ^2_X(2\mathbb N)^{p\alpha }<\infty \right\} \quad \text {and}\\ X\otimes (S)_{-1,-p} &{}= \left\{ f: \Vert f\Vert ^2_{X\otimes (S)_{-1,-p}} = \sum \limits _{\alpha \in \mathcal I}\Vert f_\alpha \Vert ^2_X(2\mathbb N)^{-p\alpha }<\infty \right\} , \end{array} \end{aligned}$$

where *X* denotes an arbitrary Banach space (both possibilities $X=\tilde{X}$ and $X=\tilde{X}'$ are allowed).

##### Definition 1

*Generalized stochastic processes* and *test stochastic processes* in Kondratiev sense are elements of the spaces respectively

$$\begin{aligned} X\otimes (S)_{-1} = \bigcup _{p\in \mathbb N} X\otimes (S)_{-1,-p} \quad \text {and} \quad X\otimes (S)_{1} = \bigcap _{p\in \mathbb N} X\otimes (S)_{1,p} . \end{aligned}$$

In this case the symbol $\otimes$ denotes the projective tensor product of two spaces, i.e., $\tilde{X}'\otimes (S)_{-1}$ is the completion of the tensor product with respect to the $\pi$-topology.

##### Remark 3

From the nuclearity of the Kondratiev space $$ (S)_{1}$$ it follows that $$(\tilde{X}\otimes (S)_{1})'\cong \tilde{X}'\otimes (S)_{-1}$$. Moreover, $$\tilde{X}'\otimes (S)_{-1}$$ is isomorphic to the space of linear bounded mappings $$\tilde{X}\rightarrow (S)_{-1}$$, and it is also isomporphic to the space of linear bounded mappings $$(S)_{1}\rightarrow \tilde{X}'$$. More details can be found in [28, 32, 39].

Throughout the paper we consider generalized stochastic processes *u* which belong to $X\otimes (S)_{-1}$ and are given by the chaos expansion form

9 $$\begin{aligned} u =\sum \limits _{\alpha \in \mathcal I}u_\alpha \otimes {H_\alpha } = u_{\mathbf {0}}(t) + \sum _{k\in \mathbb N} \, u_{\varepsilon ^{(k)}} \otimes H_{\varepsilon ^{(k)}} + \sum \limits _{|\alpha |>1} \, u_{\alpha } \otimes H_{\alpha }. \end{aligned}$$

Therein, the coefficients $u_\alpha \in X$ satisfy for some $p\in \mathbb N_0$ the convergence condition

$$\begin{aligned} \Vert u\Vert ^2_{X\otimes (S)_{-1, -p}} = \sum \limits _{\alpha \in \mathcal I} \, \Vert u_\alpha \Vert ^2_{X} \, (2\mathbb N)^{-p\alpha } < \infty . \end{aligned}$$

The value *p* corresponds to the *level of singularity* of the process *u*. Note that the deterministic part of *u* in (9) is the coefficient $u_{\mathbf {0}}$, which represents the generalized expectation of *u*. In the applications of fluid flows, the space *X* equals one of the Sobolev-Bochner spaces $L^2(T;\mathcal {V} )$ or $L^2(T;\mathcal {Q} )$.

##### Example 4

If $X=L^2(\mathbb R)$, then $u\in L^2(\mathbb R) \otimes L^2(\Omega )$ is given in the chaos expansion form $u(t, \omega ) = \sum _{\alpha \in \mathcal I} \, u_\alpha (t) \, H_\alpha (\omega )$, $t\in \mathbb R$, $\omega \in \Omega$ such that

$$\begin{aligned} \Vert u\Vert ^2_{L^2(\mathbb R) \otimes L^2(\Omega ) } = \sum _{\alpha \in \mathcal I} \, \alpha ! \, \Vert u_\alpha \Vert ^2_{L^2(\mathbb R)} \, = \sum _{\alpha \in \mathcal I} \, \int _{\mathbb R} \, \alpha ! \, |u_\alpha (t)|^2 \ \text {d}t \, < \infty . \end{aligned}$$

Stochastic processes which are elements of the space $X\otimes S'(\mathbb R) \otimes (S)_{-1} = \bigcup _{p, l\in \mathbb N} X\otimes S_{-l}(\mathbb R) \otimes (S)_{-1,-p}$ are defined similarly, cf. [27–29, 31]. More precisely, $F\in X\otimes S'(\mathbb R) \otimes (S)_{-1}$ has a chaos expansion representation

10 $$\begin{aligned} F = \sum \limits _{\alpha \in \mathcal {I}}\sum \limits _{k\in \mathbb {N}}a_{\alpha ,k}\otimes \xi _k\otimes H_{\alpha } = \sum \limits _{\alpha \in \mathcal {I}}b_\alpha \otimes H_{\alpha } = \sum \limits _{k\in \mathbb {N}}c_k\otimes \xi _k, \end{aligned}$$

where $b_\alpha =\sum _{k\in \mathbb {N}}a_{\alpha ,k}\otimes \xi _k\, \in X\otimes S'(\mathbb {R})$, $c_k =\sum _{\alpha \in \mathcal {I}}a_{\alpha ,k}\otimes H_\alpha \in X\otimes (S)_{-1}$, and $a_{\alpha ,k}\in X$. Thus, for some $p, l\in \mathbb {N}_0$, it holds that

$$\begin{aligned} \Vert F\Vert _{X \otimes \, S_{-l}(\mathbb {R})\otimes (S)_{-1, -p}}^2=\sum \limits _{\alpha \in \mathcal {I}}\sum \limits _{k\in \mathbb {N}}\Vert a_{\alpha ,k}\Vert ^2_X\, (2k)^{-l} (2\mathbb {N})^{-p\alpha } <\infty . \end{aligned}$$

The generalized expectation of *F* is the zero-th coefficient in the expansion representation (10), i.e., it is given by $\sum _{k\in \mathbb {N}} a_{\mathbf {0}, k} \otimes \xi _k = b_{\mathbf {0}}$.

Space of processes with finite second moments and square integrable trajectories $X\otimes L^2(\mathbb R)\otimes (L)^2$. It is isomporphic to $X\otimes L^2(\mathbb R\times \Omega )$ and if *X* is a separable Hilbert space, then it is also isomorphic to $L^2(\mathbb R \times \Omega , X)$.

##### Example 5

Consider $X=C^k (T)$, $k\in \mathbb N$, where *T* denotes a time interval. From the nuclearity of $(S)_{1}$ and the arguments provided in Remark 3 it follows that $C^k(T;(S)_{-1})=C^k (T) \otimes (S)_{-1}$, i.e., differentiation of a stochastic process can be carried out componentwise in the chaos expansion, cf. [28, 32]. This means that a stochastic process $u(t,\omega )$ is *k* times continuously differentiable if and only if all of its coefficients $u_\alpha$, $\alpha \in \mathcal I$ are in $C^k (T)$. The same holds for Banach space valued stochastic processes, i.e., for elements of $C^k(T;X) \otimes (S)_{-1}$, where *X* is an arbitrary Banach space. These processes can be regarded as elements of the tensor product space

$$\begin{aligned} C^k(T;X\otimes (S)_{-1}) = C^k(T;X)\otimes (S)_{-1} = \bigcup _{p=0}^{\infty }C^k(T;X)\otimes (S)_{-1,-p}. \end{aligned}$$

Since we consider weak solutions, i.e., solutions in Sobolev-Bochner spaces such as $L^2(T;X)$, it also holds $L^2(T;X\otimes (S)_{-1}) = L^2(T;X)\otimes (S)_{-1}$, as well as $H^1(T;X\otimes (S)_{-1}) = H^1(T;X)\otimes (S)_{-1}.$

In this way, by representing stochastic processes in their polynomial chaos expansion form, we are able to separate the deterministic component from the randomness of the process.

##### Example 6

*Brownian motion* $B_t(\omega ) := \langle \omega , \chi _{[0, t]} \rangle$, $\omega \in S'(\mathbb R)$, $t\ge 0$ is defined by passing though the limit in $L^2(\mathbb R)$, where $\chi _{[0,t]}$ is the characteristic function on [0, *t*]. The chaos expansion representation has the form

$$\begin{aligned} B_t (\omega ) = \sum _{k\in \mathbb N} \, \int _0^t \, \xi _k(s) \ \text {d}s \, \, H_{\varepsilon ^{(k)}} (\omega ). \end{aligned}$$

Note that for fixed *t*, $B_t$ is an element of $L^2(\Omega )$. Brownian motion is a Gaussian process with zero expectation and the covariance function $E(B_t(\omega ) B_s(\omega )) = \min \{t,s \}$. Furthermore, almost all trajectories are continuous, but nowhere differentiable functions.

*Singular white noise* is defined by the formal chaos expansion

11 $$\begin{aligned} W_{t}(\omega )=\sum \limits _{k=1}^{\infty }\xi _{k}(t){H}_{\varepsilon ^{(k)}}(\omega ), \end{aligned}$$

and is an element of the space $C^\infty (\mathbb {R}) \otimes (S)_{-1,-p}$ for $p> 1$, cf. [22]. With weak derivatives in the $(S)_{-1}$ sense, it holds that $\frac{\text {d}}{\text {d}t} B_{t}=W_{t}$. Both, Brownian motion and singular white noise, are Gaussian processes and have chaos expansion representations via Fourier-Hermite polynomials with multi-indeces of length one, i.e., belong to the Wiener chaos space of order one.

More general, the chaos expansion of a Gaussian process $G_t$ in $S'(\mathbb R) \otimes (S)_{-1}$, which belongs to the Wiener chaos space of order one, is given by

12 $$\begin{aligned} G_t(\omega ) =\sum \limits _{k=1}^{\infty } m_k(t) {H}_{\varepsilon ^{(k)}}(\omega ) = \sum \limits _{k=1}^{\infty } \, \sum \limits _{n=1}^{\infty } m_{kn}\, \xi _n(t) {H}_{\varepsilon ^{(k)}}(\omega ) , \end{aligned}$$

with coefficients $m_k$ being deterministic generalized functions and $m_{kn}\in \mathbb R$ such that the condition

$$\begin{aligned} \sum \limits _{k=1}^{\infty } \Vert m_k\Vert _{-l}^2 (2 k)^{-p} = \sum \limits _{k=1}^{\infty } \sum \limits _{n=1}^{\infty } m_{kn}^2 \, (2n)^{-l} \, (2k)^{-p} < \infty \end{aligned}$$

holds for some $l, p\in \mathbb N_0$. One can also consider a generalized Gaussian process $G\in X \otimes (S)_{-1}$ with a Banach space *X* of the form

$$\begin{aligned} G =\sum _{k=1}^{\infty } m_k \, {H}_{\varepsilon ^{(k)}}, \end{aligned}$$

with coefficients $m_k \in X$ that satisfy

13 $$\begin{aligned} \sum \limits _{k=1}^{\infty } \Vert m_k\Vert _{X}^2 (2 k)^{-p} < \infty , \end{aligned}$$

For example, in Sect. 3.3 we deal with $X= L^2(T;\mathcal {V} ^*)$.

The Wick product of two stochastic processes is defined in an analogue way as it was defined for random variables in (8) and generalized random variables [30]. Let *F* and *G* be stochastic processes given in their chaos expansion forms $F= \sum _{\alpha \in \mathcal I} f_\alpha \, \otimes \, H_\alpha$ and $G= \sum _{\alpha \in \mathcal I} g_\alpha \,\otimes \, H_\alpha$, $f_\alpha , g_\alpha \in X$ for all $\alpha \in \mathcal I$. Assuming that $f_\alpha \, g_\beta \in X$, for all $\alpha , \beta \in \mathcal I$, the *Wick product* $F\lozenge G$ is defined by

14 $$\begin{aligned} F\lozenge G \, =\, \sum _{\gamma \in \mathcal I}\left( \sum _{\alpha +\beta =\gamma }f_\alpha g_\beta \right) \otimes H_\gamma . \end{aligned}$$

The examples considered in this paper use either $X=L^2(T;\mathcal {V} ^*)$ or $X= C^k(T)$. The space of stochastic processes $X\otimes (S)_{-1}$ is closed under the Wick multiplication. This is stated in the following theorem. The proof can be found in [28].

##### Theorem 5

([28]) Consider $F\in X\otimes (S)_{-1, -p_1}$ and $G\in X\otimes (S)_{-1, -p_2}$ for some $p_1, p_2\in \mathbb N_0$. Then the Wick product $F\lozenge G$ is a well-defined element in the space $X\otimes (S)_{-1, -q}$ for $q\ge p_1+p_2 +2$.

#### Coordinatewise operators

We follow the classification of stochastic operators given in [32] and consider the following two classes. We say that an operator $\mathcal A$ defined on $X\otimes (S)_{-1}$ is a *coordinatewise operator* if it is composed of a family of operators $\{A_\alpha \}_{\alpha \in \mathcal I}$, $A_\alpha : X \rightarrow X$, $\alpha \in \mathcal I$, such that for a process $u= \sum _{\alpha \in \mathcal I} \, u_\alpha \, \otimes \, H_\alpha \in X\otimes (S)_{-1}$, $u_\alpha \in X$, $\alpha \in \mathcal I$ it holds that

$$\begin{aligned} \mathcal A u = \sum _{\alpha \in \mathcal I} \, A_\alpha u_\alpha \, \otimes \, H_\alpha \, . \end{aligned}$$

If $A_\alpha = A$ for all $\alpha \in \mathcal I$, then the operator $\mathcal A$ is called a *simple coordinatewise operator*.

### Chaos expansion approach

We return to the stochastic operator DAEs (6 and (7 where the noise terms are generalized Gaussian stochastic processes as given in (12). Within the next two subsections, we consider the influence of these perturbations. Applying the chaos expansion method, we transform the stochastic systems into deterministic problems, which we solve by induction over the length of the multi-index $\alpha$. Clearly, we represent all the processes appearing in the stochastic equation by their chaos expansion forms and, since the representation in the Fourier-Hermite polynomial basis is unique, equalize the coefficients. In this section, we assume $\mathcal K$ and $\mathcal B$ to be simple coordinatewise operators, i.e., for $u=\sum _{\alpha \in \mathcal I}u_\alpha \otimes H_\alpha$ we have

15 $$\begin{aligned} \mathcal {K} u = \sum _{\alpha \in \mathcal I}K u_\alpha \otimes H_\alpha \quad \text {and}\quad \mathcal {B} u = \sum _{\alpha \in \mathcal I}B u_\alpha \otimes H_\alpha . \end{aligned}$$

Note that this implies that $\mathcal B^*$ is a simple coordinatewise operator as well. A more general case of coordinatewise operators is considered in Sect. 4. In the following, we assume that *K* and *B* are linear and that they satisfy Assumption 1. For the right-hand side of the differential equation (6a), namely stochastic process $\mathcal {F}$, and the constraint (6b), namely stochastic process $\mathcal {G}$, we assume that they are given in the chaos expansion forms

16 $$\begin{aligned} \mathcal {F} = \sum _{\alpha \in \mathcal I} \, f_\alpha \otimes H_{\alpha } \quad \text {and}\quad \mathcal {G} = \sum _{\alpha \in \mathcal I} \, g_\alpha \otimes H_{\alpha }. \end{aligned}$$

Therein, corresponding to the deterministic setting of Sect. 2.1, the deterministic coefficients satisfy $f_\alpha \in L^2(T;\mathcal {V} ^*)$ and $g_\alpha \in H^1(T;\mathcal {Q} ^*)$. Furthermore, we assume that for some positive *p* it holds that

17 $$\begin{aligned} \sum _{\alpha \in \mathcal I} \Vert f_\alpha \Vert _{L^2(\mathcal {V} ^*)}^2 (2\mathbb N)^{-p\alpha }<\infty \quad \text {and}\quad \sum _{\alpha \in \mathcal I} \Vert g_\alpha \Vert _{H^1(\mathcal {Q} ^*)}^2 (2\mathbb N)^{-p\alpha } <\infty . \end{aligned}$$

#### Remark 4

Since the family of spaces $$(S)_{-1, -p} $$ is monotone, i.e., it holds that $$(S)_{-1, - p_1} \subset (S)_{-1, - p} $$ for $$p_1 < p$$, we may assume in (17) that all the convergence conditions hold for the same level of singularity *p*. Clearly, for two different $$p_1$$ and $$p_2$$ we can take *p* to be $$p=\max \{p_1, p_2\}$$ and thus, obtain that generalized stochastic processes satisfies (17) in the biggest space $$(S)_{-1,-p}$$. In that sense, we use in the sequel always the same level of singularity *p*.

We seek for solutions *u* and $\lambda$ of stochastic operator DAEs (6 and (7, which are *stochastic processes* belonging to $L^2(\mathcal {V} )\otimes (S)_{-1}$ and $L^2(\mathcal {Q} )\otimes (S)_{-1}$, respectively. Their chaos expansions are given by

18 $$\begin{aligned} u =\sum \limits _{\alpha \in \mathcal I}u_\alpha \otimes {H_\alpha } \quad \text {and}\quad \lambda = \sum _{\alpha \in \mathcal I} \, \lambda _\alpha \otimes H_{\alpha }. \end{aligned}$$

The aim is to calculate the unknown coefficients $u_\alpha$ and $\lambda _\alpha$ for all $\alpha \in \mathcal I$, which then give the overall solutions *u* and $\lambda$. Furthermore, we are going to prove bounds on the solutions, provided that the stated assumptions on the given processes $\mathcal F$ and $\mathcal G$, the initial condition, and the noise terms are fulfilled.

Considering the stochastic operator DAE equations (6 and (7, we apply at first the chaos expansion method to the initial condition $$ u(0) = u^0$$ and obtain

$$\begin{aligned} u^0 = \sum _{\alpha \in \mathcal I} \, u_\alpha (0) \, H_\alpha \, = \, \sum _{\alpha \in \mathcal I} \, u_\alpha ^0 \, H_\alpha . \end{aligned}$$

Thus, the initial condition reduces to the family of conditions $u_\alpha (0) = u^0_\alpha \in \mathcal {H}$ for every $\alpha \in \mathcal I$. In order to achieve consistency, the initial data has to be of the form

19 $$\begin{aligned} u^0_\alpha = u^0_{B, \alpha } + B^- g_\alpha (0) , \qquad \alpha \in \mathcal I , \end{aligned}$$

with an arbitrary $u^0_{B, \alpha }$ from the closure of the kernel of *B* in $\mathcal {H}$ and $B^-$ denoting the right-inverse of the operator *B*, cf. Remark 1.

### Noise in the differential equation

Consider the system (6 with a stochastic perturbation given in the form of a generalized Gaussian stochastic process in the Wiener chaos space of order one as in (12), i.e., we consider the initial value problem

20 $$\begin{aligned} \dot{u}(t) + \mathcal {K} u(t) + \mathcal {B}^* \lambda (t)= \mathcal {F}(t)+ G_t, \nonumber \\ \mathcal {B} u(t) = \mathcal {G}(t), \qquad u(0)=u^0=u_\mathcal {B} ^0 + \mathcal {B} ^- \mathcal {G} (0). \end{aligned}$$

#### Example 7

(Randomly forced Stokes equation) We consider the randomly forced Stokes equation, i.e. Stokes equation with noise forcing term. In this case, the operator equation (20) is equal to the weak formulation of the stochastically perturbed Stokes equations

$$\begin{aligned} \dot{u} - \Delta u + \nabla \lambda = \tilde{f}, \qquad \nabla \cdot u = 0, \qquad u(0) = u^0, \end{aligned}$$

where the flow $\tilde{f} = f + G_t$, $t\in T$, is subject to an external forcing. We refer the reader to [12] for a detailed explanation. Note that, in general, additive noise is interpreted in applications as a perturbation of the original model (in this case Example 4).

We summarize the needed requirements in the following assumption.

#### Assumption 2

1. Operators $\mathcal {K}$ and $\mathcal {B}$ are simple coordinatewise operators with corresponding deterministic operators $K:\mathcal {V} \rightarrow \mathcal {V} ^*$ and $B:\mathcal {V} \rightarrow \mathcal {Q} ^*$, which satisfy the assumptions stated in Assumption 1.
2. The stochastic processes $\mathcal {F}$ and $\mathcal {G}$ are given in their chaos expansion forms (16) such that the conditions in (17) hold.
3. The process $G_t$ is a Gaussian noise term represented in the form (12), with $m_k \in L^2(T;\mathcal {V} ^*)$, $k\in \mathbb N$, such that (13) holds.
4. The stochastic process $u^0$ has the chaos expansion form $u^0 = \sum _{\alpha \in \mathcal I} \, u_\alpha ^0 \, H_\alpha$ such that for some $p\in \mathbb N_0$ it holds that
  21 $$\begin{aligned} \sum _{\alpha \in \mathcal I} \left\| u^0_{\alpha } \right\| _\mathcal {H} ^2 (2\mathbb N)^{-p\alpha } < \infty . \end{aligned}$$

#### Remark 5

If the initial data is consistent, then Assumption 2 and equation (19) imply that condition (21) can be replaced by

22 $$\begin{aligned} \sum _{\alpha \in \mathcal I} \left\| u^0_{B,\alpha } \right\| _\mathcal {H} ^2 (2\mathbb N)^{-p\alpha } < \infty , \end{aligned}$$

with $u^0_{B,\alpha }$ given in (19).

#### Theorem 6

Let Assumption 2 be satisfied. Then, for any consistent initial data there exists a unique solution $u\in L^2(T;\mathcal {V} )\otimes (S)_{-1}$ of the stochastic DAE (20).

#### Proof

We represent all the processes in (20) in their chaos expansion forms, apply (15) and thus, reduce it to an infinite triangular system of deterministic initial value problems, which can be solved recursively over the length of multi-index $\alpha$. We obtain the system

$$\begin{aligned} \sum \limits _{\alpha \in \mathcal I} \, \big ( \dot{u}_\alpha (t) + K u_\alpha (t) + B^* \lambda _\alpha (t) \big ) \, H_\alpha (\omega )= & {} \sum \limits _{\alpha \in \mathcal I} \, f_\alpha (t) \, H_\alpha (\omega ) + \sum \limits _{k\in \mathbb N} \, m_k (t) \, H_\alpha (\omega ), \\ \sum \limits _{\alpha \in \mathcal I} \, B u_\alpha (t) \, H_\alpha (\omega )= & {} \sum \limits _{\alpha \in \mathcal I} \, g_\alpha (t) \, H_\alpha (\omega ) \end{aligned}$$

with $u(0)=u^0$, i.e., initial data with coefficients given in (19) that satisfy (21). Thus,

1. for $|\alpha | =0$, i.e., for $\alpha = \mathbf {0} = (0,0,\ldots )$, we have to solve
  23 $$\begin{aligned} \dot{u}_{\mathbf {0} }(t) + {K} u_{\mathbf {0} }(t) + {B}^* \lambda _{\mathbf {0} } (t) = f_{\mathbf {0}}(t), {B} u_{\mathbf {0} }(t) = g_{\mathbf {0}}(t), \quad u_{\mathbf {0}} = u^0_{B,\mathbf {0}} + B^- g_{\mathbf {0}}(0). \end{aligned}$$
  Note that system (23) is a deterministic problem of the form (1, where *F* and *G* from (1 are equal to $f_{\mathbf {0}}$ and $g_{\mathbf {0}}$, respectively. Moreover, the system (23) can be obtained by taking the expectation of the system (20). The assumptions on the operators and right-hand sides $f_\mathbf {0}\in L^2(T;\mathcal {V} ^*)$, $g_\mathbf {0}\in H^1(T;\mathcal {Q} ^*)$ imply the existence of a solution $u_{\mathbf {0} }$, $\lambda _{\mathbf {0}}$.
2. for $|\alpha | =1$, i.e., for $\alpha = \varepsilon ^{(k)}$, $k\in \mathbb N$, we obtain the system
  24 $$\begin{aligned} \dot{u}_{\varepsilon ^{(k)} }(t) + {K} u_{\varepsilon ^{(k)} }(t) + {B}^* \lambda _{\varepsilon ^{(k)}} (t)= f_{\varepsilon ^{(k)}} (t) + m_{k}(t), {B} u_{\varepsilon ^{(k)}}(t) = g_{\varepsilon ^{(k)}}(t) \end{aligned}$$
  with initial condition $u_{\varepsilon ^{(k)} } (0) = u^0_{B, \varepsilon ^{(k)}} + B^- g_{\varepsilon ^{(k)}} (0)$. For each $k\in \mathbb N$ system (24) is a deterministic initial value problem of the form (1, with the choice $F =f_{\varepsilon ^{(k)}} + m_k$ and $G =g_{\varepsilon ^{(k)}}$.
3. for $|\alpha |>1$, we finally solve
  25 $$\begin{aligned} \dot{u}_{\alpha }(t) + {K} u_{\alpha }(t) + {B}^* \lambda _{\alpha } (t) = f_\alpha (t), {B} u_{\alpha }(t)= g_{\alpha }(t), \quad u_{\alpha }(0) = u_{B,\alpha }^0 + B^- g_{\alpha }(0). \end{aligned}$$
  Again, system (25) is a deterministic operator DAE, which can be solved in the same manner as the system (23).

From (23) we obtain $u_{\mathbf {0}}$ and $\lambda _{\mathbf {0}}$. Further, from (24) we obtain the coefficients $u_{\alpha }$ and $\lambda _\alpha$ for $|\alpha |=1$ and from (25) the remaining coefficients. Note that all these systems may be solved in parallel.

As the last step of the analysis, we prove the convergence of the obtained solution in the space of Kondratiev generalized stochastic processes, i.e., we prove that $\Vert u\Vert ^2_{L^2(\mathcal {V} ) \otimes (S)_{-1}} < \infty$, for $u=\sum _{\alpha \in \mathcal I} \, u_\alpha \otimes H_\alpha$. More precisely we show that

$$\begin{aligned} \sum _{\alpha \in \mathcal I} \Vert u_\alpha \Vert ^2_{L^2(\mathcal {V} )} (2\mathbb N)^{- p \alpha } < \infty \end{aligned}$$

holds for some $p\in \mathbb N_0$. For this, we apply the estimate from Theorem 1 to the deterministic operator DAEs (23)–(25) for the coefficients $u_\alpha$. For $u_{\mathbf {0}}$ we obtain by Theorem 1 the estimate

26 $$\begin{aligned} \Vert u_{\mathbf {0}} \Vert _{L^2(\mathcal {V} )}^2 \lesssim \left\| u^0_{B,\mathbf {0}} \right\| _\mathcal {H} ^2 + \left\| f_\mathbf {0}\right\| ^2_{L^2(\mathcal {V} ^*)} + \left\| g_\mathbf {0}\right\| ^2_{H^1(\mathcal {Q} ^*)}. \end{aligned}$$

Similarly, for $|\alpha |=1$ and $|\alpha |>1$, we obtain respectively the estimates

$$\begin{aligned} \Vert u_{\varepsilon ^{(k)}} \Vert _{L^2(\mathcal {V} )}^2&\lesssim \left\| u^0_{B,\varepsilon ^{(k)}}\right\| _\mathcal {H} ^2 + \left\| f_{\varepsilon ^{(k)}} + m_k\right\| ^2_{L^2(\mathcal {V} ^*)} + \Vert g_{\varepsilon ^{(k)}}\Vert ^2_{H^1(\mathcal {Q} ^*)}, \, \, k\in \mathbb N \, \, \text {and}\\ \Vert u_{\alpha } \Vert _{L^2(\mathcal {V} )}^2&\lesssim \left\| u^0_{B,\alpha }\right\| _\mathcal {H} ^2 + \Vert f_\alpha \Vert ^2_{L^2(\mathcal {V} ^*)} + \Vert g_\alpha \Vert ^2_{H^1(\mathcal {Q} ^*)} , \quad |\alpha |>1. \end{aligned}$$

Note that the involved constants are equal for all estimates, since we have assumed simple coordinatewise operators. Summarizing the results, we obtain

$$\begin{aligned} \sum _{\alpha \in \mathcal I} \Vert u_\alpha \Vert ^2_{L^2(\mathcal {V} )} (2\mathbb N)^{- p\alpha }&\lesssim \sum _{\alpha \in \mathcal I} \left\| u^0_{B,\alpha } \right\| _\mathcal {H} ^2 (2\mathbb N)^{-p\alpha } + \sum _{\alpha \in \mathcal I} \Vert f_\alpha \Vert ^2_{L^2(\mathcal {V} ^*)} (2\mathbb N)^{-p\alpha } \\&\quad + \sum _{k=1}^\infty \Vert m_k \Vert ^2_{L^2(\mathcal {V} ^*)} (2k)^{-p} \!+\! \sum _{\alpha \in \mathcal I} \Vert g_\alpha \Vert ^2_{H^1(\mathcal {Q} ^*)} (2\mathbb N)^{-p\alpha } < \infty , \end{aligned}$$

where we have used the linearity, the triangular inequality, and the relation $(2\mathbb N)^{\varepsilon ^{(k)}} = 2k$, $k\in \mathbb N$. The assumptions (13), (17), and (21) show that the right-hand side is bounded and thus, completes the proof. $\square$

#### Remark 6

If the process $\mathcal F$ in (20) is a deterministic function, then it can be represented by $\mathcal F = f_{\mathbf 0}$, since the remaining coefficients satisfy $f_\alpha =0$ for all $|\alpha |>0$. Therefore, systems (24) and (25) further simplify.

As mentioned in Remark 2, a similar result can be formulated for the Lagrange multiplier if we assume stronger regularity assumptions. For completeness we state the following result for the Lagrange multiplier but leave out the proof.

#### Theorem 7

Let Assumption 2 be satisfied. Assume additionally $f_\alpha \in L^2(T;\mathcal {H} ^*)$ and $u^0_{B,\alpha } \in \mathcal {V}$ and let the operator *K* be symmetric. Then, for any consistent initial data there exists a unique Lagrange multiplier $\lambda \in L^2(T;\mathcal {Q} )\otimes (S)_{-1}$ of the stochastic operator DAE (20).

One may also consider stochastic operator DAEs (20) which include a more general form of the Gaussian noise $G_t$, i.e.,

27 $$\begin{aligned} G_t (\omega ) = \sum _{|\alpha | > 0} \, m_\alpha (t) \, H_\alpha (\omega ), \end{aligned}$$

where $G_t$ has also non-zero coefficients of order greater than one. The solution for this case can be provided similarly to the presented case for Gaussian noise in the Wiener chaos space of order one.

#### Theorem 8

Let the assumptions 1, 2 and 4 from Assumption 2 hold and let the process $G_t$ be a Gaussian process of the form (27) such that for some $p\ge 0$ it holds that

$$\begin{aligned} \sum _{|\alpha | > 0} \, \Vert m_\alpha \Vert ^2_{L^2(\nu ^*)} \, (2\mathbb N)^{-p\alpha } \, < \infty . \end{aligned}$$

Then, for any consistent initial data that satisfies (21) the stochastic operator DAE (20) has a unique solution $u\in L^2(T;\mathcal {V} )\otimes (S)_{-1}$.

#### Proof

The system of deterministic DAEs obtained from (20) by applying the chaos expansion method contains (23) for $|\alpha |=0$ and

28 $$\begin{aligned} \dot{u}_{\alpha }(t) + {K} u_{\alpha }(t) + {B}^* \lambda _{\alpha } (t)= & {} f_\alpha (t) + m_\alpha (t), \nonumber \\ {B} u_{\alpha }(t)= & {} g_{\alpha }(t), \end{aligned}$$

with the condition $u_{\alpha }(0) = u_{B,\alpha }^0 + B^- g_{\alpha }(0)$, for $|\alpha |>0$. By solving the obtained systems, we obtain the unknown coefficients $u_\alpha$, $\alpha \in \mathcal I$. By applying Theorem 1, we obtain the estimates (26) for $|\alpha |=0$ and

$$\begin{aligned} \Vert u_{\alpha } \Vert _{L^2(\mathcal {V} )}^2 \lesssim \left\| u^0_{B,\alpha }\right\| _\mathcal {H} ^2 + \Vert f_\alpha + m_\alpha \Vert ^2_{L^2(\mathcal {V} ^*)} + \Vert g_\alpha \Vert ^2_{H^1(\mathcal {Q} ^*)} \end{aligned}$$

for $|\alpha |>0$. Hence, the solution $u=\sum _{\alpha \in \mathcal I} u_\alpha H_\alpha$ satisfies the estimate

29 $$\begin{aligned} \sum \limits _{\alpha \in \mathcal I} \Vert u_\alpha \Vert ^2_{L^2(\mathcal {V} )} (2\mathbb N)^{- p\alpha }\lesssim & {} \sum \limits _{\alpha \in \mathcal I} \left\| u^0_{B,\alpha } \right\| _\mathcal {H} ^2 (2\mathbb N)^{-p\alpha } + \sum \limits _{\alpha \in \mathcal I} \Vert f_\alpha \Vert ^2_{L^2(\mathcal {V} ^*)} (2\mathbb N)^{-p\alpha } \nonumber \\&+ \sum \limits _{|\alpha |>0}^\infty \Vert m_\alpha \Vert ^2_{L^2(\mathcal {V} ^*)} (2\mathbb N)^{-p\alpha } + \sum \limits _{\alpha \in \mathcal I} \Vert g_\alpha \Vert ^2_{H^1(\mathcal {Q} ^*)} (2\mathbb N)^{-p\alpha } < \infty .\qquad \end{aligned}$$

This shows that *u* belongs to $L^2(T;\mathcal {V} )\otimes (S)_{-1}$. $\square$

#### Remark 7

Let the assumptions of Theorem 8 hold. If we assume additionally that $f_\alpha \in L^2(T;\mathcal {H} ^*)$, $u^0_{B,\alpha } \in \mathcal {V}$ and the operator *K* is symmetric, then there exists a unique Lagrange multiplier $\lambda \in L^2(T;\mathcal {Q} )\otimes (S)_{-1}$ of the stochastic operator DAE (20).

### Noise in the constraint equation

Consider the stochastic operator DAE (7, where the noise terms are given in the form of two Gaussian white noise processes $G_t$ and $G_t^{(1)}$ belonging to the Wiener chaos space of order one. More precisely, we consider the initial value problem

30 $$\begin{aligned} \begin{array}{cccccccccc} \dot{u}(t) &{} + &{} \mathcal {K} u(t) &{} +&{} \mathcal {B}^* \lambda (t) &{} = &{} \mathcal {F}(t) + G_t, \\ &{} &{} \mathcal {B} u(t) &{} &{} &{} = &{} \mathcal {G}(t) + G_t^{(1)} \end{array} \end{aligned}$$

with the initial condition $u(0)=u^0$. Note that the initial data $u^0$ has to be consistent again. Here, the consistency condition includes the perturbation $G_t^{(1)}$ such that the consistent initial data of the unperturbed problem may not be consistent in this case. We assume

31 $$\begin{aligned} G_t^{(1)} (\omega )= \sum _{k=1}^\infty \, m^{(1)}_k(t) \, H_{\varepsilon ^{(k)} } (\omega ), \end{aligned}$$

where $m^{(1)}_k \in L^2(T;\mathcal {Q} ^*)$. We still keep the Assumption 2 for the operators $\mathcal K$ and $\mathcal B$, processes $\mathcal F$ and $\mathcal G$ and Gaussian noise $G_t$. Note that $m_k \in L^2(T;\mathcal {V} ^*)$, $k\in \mathbb N$. Then, system (30) reduces to the following deterministic systems:

1. for $|\alpha | =0$, i.e., for $\alpha = \mathbf {0} = (0,0,\ldots )$, we obtain
  32 $$\begin{aligned} \dot{u}_{\mathbf {0} }(t) + {K} u_{\mathbf {0} }(t) + {B}^* \lambda _{\mathbf {0} } (t)= f_{\mathbf {0}}(t), {B} u_{\mathbf {0} }(t) = g_{\mathbf {0}}(t), \quad u_{\mathbf {0}} = u^0_{\mathbf {0}}. \end{aligned}$$
2. for $|\alpha | =1$, i.e., for $\alpha = \varepsilon ^{(k)}$, $k\in \mathbb N$, we have
  33 $$\begin{aligned} \dot{u}_{\varepsilon ^{(k)} }(t) + {K} u_{\varepsilon ^{(k)} }(t) + {B}^* \lambda _{\varepsilon ^{(k)} } (t)= & {} f_{\varepsilon ^{(k)}}(t) + m_k (t),\nonumber \\ {B} u_{\varepsilon ^{(k)}}(t)= & {} g_{\varepsilon ^{(k)}}(t) + m_k^{(1)}(t), \nonumber \\ u_{\varepsilon ^{(k)} } (0)= & {} u^0_{\varepsilon ^{(k)}}. \end{aligned}$$
3. for $|\alpha |>1$, we solve
  34 $$\begin{aligned} \dot{u}_{\alpha }(t) + {K} u_{\alpha }(t) + {B}^* \lambda _{\alpha } (t)= & {} f_\alpha (t), \nonumber \\ {B} u_{\alpha }(t)= & {} g_{\alpha }(t),\nonumber \\ u_{\alpha }(0) = u_{\alpha }^0. \end{aligned}$$

We emphasize that the operator DAEs (32)–(34) can be solved in parallel again. However, system (33) is deterministic with a perturbation in the constraint, cf. Sect. 2.2 with $\theta = m_k^{(1)}$. The estimate (3) shows that this results in instabilities such that the stochastic truncation cannot converge. To see this, note that a computation as in the proof of Theorem 6 includes terms of the form $\Vert \dot{m}_k^{(1)} \Vert _{L^2(\mathcal {Q} ^*)}$. Thus, the assumed boundedness

$$\begin{aligned} \sum \limits _{k=1}^{\infty } \left\| m_k^{(1)} \right\| _{L^2(\mathcal {Q} ^*)} (2 k)^{-p} < \infty , \end{aligned}$$

is not sufficient to bound the terms which involve the derivatives of $m_k^{(1)}$. Consequently, we have to consider the regularized formulation.

### Regularization

We have seen that the solution is very sensitive to perturbations in the constraint equation. As for the deterministic case in Sect. 2.3, we need a regularization. The extended (but equivalent) system to (30) with stochastic noise terms, has the form

35a $$\begin{aligned} {\dot{u}_{1}}(t) + v_2(t) + {\mathcal {K} {\big (u_1(t)+u_2(t)\big )}} + {\mathcal {B} }^{*} {\lambda (t)}&= {\mathcal {F} (t)} + G_t \end{aligned}$$

35b $$\begin{aligned} {\mathcal {B} } u_2(t)&= {\mathcal {G} (t)} + G_t^{(1)} \end{aligned}$$

35c $$\begin{aligned} {\mathcal {B} } v_2(t)&= {\dot{\mathcal {G} }}(t) + G_t^{(2)}. \end{aligned}$$

Note that, because of the extension of the system, we consider another perturbation $G_t^{(2)}$ in (35, represented in the form

36 $$\begin{aligned} G_t^{(2)} (\omega )= \sum _{k=1}^\infty \, m^{(2)}_k(t) \, H_{\varepsilon ^{(k)} } (\omega ). \end{aligned}$$

The chaos expansion approach leads again to a system of deterministic operator DAEs. Since the perturbations have zero mean and are of order one only, we only consider the case with $\alpha = \varepsilon ^{(k)}$, which leads to

37 $$\begin{aligned} \dot{u}_{1,\varepsilon ^{(k)} }(t) + v_{2,\varepsilon ^{(k)} }(t) + {K}\big (u_{1,\varepsilon ^{(k)} } + u_{2,\varepsilon ^{(k)} }\big )(t)+ {B}^* \lambda _{\varepsilon ^{(k)} } (t)&= f_{\varepsilon ^{(k)}}(t) + m_k(t), \nonumber \\ {B} u_{2,\varepsilon ^{(k)}}(t)&= g_{\varepsilon ^{(k)}}(t) + m_k^{(1)}(t), \nonumber \\ {B} v_{2,\varepsilon ^{(k)}}(t)&= \dot{g}_{\varepsilon ^{(k)}}(t) + m_k^{(2)}(t). \end{aligned}$$

Note here that the obtained system (37) corresponds to the perturbed extended system with perturbations in the constraint equation, i.e., it corresponds to the system

38 $$\begin{aligned} \dot{u}_{1,\varepsilon ^{(k)} }(t) + v_{2,\varepsilon ^{(k)}}(t) + {K}\big (u_{1,\varepsilon ^{(k)} }+ u_{2,\varepsilon ^{(k)} }\big )(t)+ {B}^* \lambda _{\varepsilon ^{(k)} } (t)&= f_{\varepsilon ^{(k)}}(t) + m_k(t), \nonumber \\ {B} u_{2,\varepsilon ^{(k)}}(t)&= g_{\varepsilon ^{(k)}}(t),\nonumber \\ \quad {B} v_{2,\varepsilon ^{(k)}}(t)&= \dot{g}_{\varepsilon ^{(k)}}(t), \end{aligned}$$

that is equivalent to (24). Therefore, the stochastic operator DAE (30) can be treated as perturbed stochastic operator DAE (20) with the perturbation appearing in its constraint equation.

Recall that the formulation (37) allows an estimate of the coefficients $u_{1,\varepsilon ^{(k)}}$ without the derivatives of the perturbations, cf. estimate (5). This then leads to a uniform bound of the solution $u_1$, $u_2$, similarly as in Theorem 6. Furthermore, the regularization solves the problem of finding consistent initial data. Here, the condition reads $u_{1,\varepsilon ^{(k)} } (0) = u^0_{1, \varepsilon ^{(k)}}$ and thus, does not depend on the perturbations. Finally, Theorem 9 summarizes the discussion. Therein we use the following notation. We denote by $(u_1, u_2, v_2, \lambda _2)$ the solution of

39a $$\begin{aligned} {\dot{u}_1}(t) + v_2(t) + {\mathcal {K} {\big (u_1(t)+u_2(t)\big )}} + {\mathcal {B} }^{*} {\lambda } (t)&= {\mathcal {F} (t)} + G_t \end{aligned}$$

39b $$\begin{aligned} {\mathcal {B} u_2}(t)&= {\mathcal {G} (t)} \end{aligned}$$

39c $$\begin{aligned} {\mathcal {B} } v_2(t)&= {\dot{\mathcal {G} }}(t) \end{aligned}$$

and by $(\hat{u}_1, \hat{u}_2, \hat{v}_2, \hat{\lambda }_2)$ the solution of its perturbed operator DAE (35, while by $\mathbf {e}_1$ we denote the error in $u_1$, i.e. $\mathbf {e}_1 = \hat{u}_1 - u_1$.

#### Theorem 9

Let the Assumption 2 hold. Consider the perturbations $G_t^{(1)}$ and $G_t^{(2)}$ of the right hand sides of the operator DAE (39 that are of the forms (31) and (36), with the coefficients $m^{(1)}_k \in L^2(T;\mathcal {Q} ^*)$ and $m^{(2)}_k \in L^2(T;\mathcal {Q} ^*)$ such that

40 $$\begin{aligned} \sum \limits _{k=1}^{\infty } \left\| m^{(1)}_k\right\| _{L^2(\mathcal {Q} ^*)}^2 (2 k)^{-p}< \infty \quad \text {and} \quad \sum \limits _{k=1}^{\infty } \left\| m^{(2)}_k\right\| _{L^2(\mathcal {Q} ^*)}^2 (2 k)^{-p} < \infty \end{aligned}$$

for some $p\in \mathbb N_0$. Then, the error $\mathbf {e}_1$ satisfies the following estimate

41 $$\begin{aligned} \begin{array}{ll} &{}\Vert \mathbf {e}_1\Vert ^2_{C(T;\mathcal H) \otimes (S)_{-1, -p}} + \Vert \mathbf {e}_1\Vert ^2_{L^2(\mathcal {V} ) \otimes (S)_{-1, -p}} \\ &{}\qquad \qquad \lesssim \sum \limits _{k\in \mathbb N}\, \left\| m^{(1)}_k \right\| _{L^2(\mathcal {Q} ^*)} (2k)^{-p} + \sum \limits _{k\in \mathbb N}\, \left\| m^{(2)}_k \right\| _{L^2(\mathcal {Q} ^*)} (2k)^{-p} < \infty . \end{array} \end{aligned}$$

#### Proof

After applying the chaos expansion method to the extended operator DAE (39 we obtain the system of deterministic problems, i.e. for $|\alpha |=1$ we obtain (38), while for all $|\alpha |\not =1$ we obtain

42 $$\begin{aligned} \dot{u}_{1,\alpha }(t) + v_{2,\alpha }(t) + {K}\big (u_{1,\alpha }(t) + u_{2,\alpha }(t) \big )+ {B}^* \lambda _{\alpha } (t)= & {} f_{\alpha }(t) , \nonumber \\ {B} u_{2,\alpha }(t)= & {} g_{\alpha }(t), \nonumber \\ \quad {B} v_{2,\alpha }(t)= & {} \dot{g}_{\alpha }(t). \end{aligned}$$

On the other hand, by applying the chaos expansion method to the extended operator DAE (35 we obtain the deterministic systems, i.e. for $|\alpha |=1$ we obtain (37) and for $|\alpha |\not =1$ the system (42).

The difference between the systems (38)–(42) of the original problem (39 and the system (37)–(42) of the perturbed problem (35 is seen only for $\alpha =\varepsilon ^{(k)}$, $k\in \mathbb N$. Thus, only nonzero coefficients of the error $\mathbf {e}_1$ are obtained for $\alpha =\varepsilon ^{(k)}$, $k\in \mathbb N$, i.e., $\mathbf {e}_{1, \varepsilon ^{(k)}} = \hat{u}_{1, \varepsilon ^{(k)}} - u_{1, \varepsilon ^{(k)}}$ and $\mathbf {e}_{1, \alpha } = \hat{u}_{1, \alpha } - u_{1, \alpha } =0$, for $|\alpha |\not =1$. Similarly, the remaining nonzero errors are $\mathbf {e}_{2, \varepsilon ^{(k)}} = \hat{u}_{2, \varepsilon ^{(k)}} - u_{2, \varepsilon ^{(k)}}$, $\mathbf {e}_{v, \varepsilon ^{(k)}} = \hat{v}_{ \varepsilon ^{(k)}} - v_{ \varepsilon ^{(k)}}$ and $\mathbf {e}_{\lambda , \varepsilon ^{(k)}} = \hat{\lambda }_{ \varepsilon ^{(k)}} - \lambda _{\varepsilon ^{(k)}}$. Moreover, the notation of Theorem 2, we have $\delta _{ \varepsilon ^{(k)}}(t) =0$, $\theta _{ \varepsilon ^{(k)}} = m_k^{(1)} \in L^2(T;\mathcal {Q} ^*)$ and $\xi _{ \varepsilon ^{(k)}}(t) = m_k^{(2)}\in L^2(T;\mathcal {Q} ^*)$, $k\in \mathbb N$ and $\mathbf {e}_{1, 0} = 0$. Therefore, we apply Theorem 2 and obtain the estimates

43 $$\begin{aligned} \left\| \mathbf {e}_{1, \varepsilon ^{(k)}} \right\| ^2_{C(T;\mathcal H)} + \left\| \mathbf {e}_{1, \varepsilon ^{(k)}} \right\| ^2_{L^2(T;\mathcal {V} )} \lesssim \left\| m_k^{(1)}\right\| ^2_{L^2(\mathcal {Q} ^*)} + \left\| m_k^{(1)}\right\| ^2_{L^2(\mathcal {Q} ^*)}, \end{aligned}$$

for all $k\in \mathbb N$. Since it holds

$$\begin{aligned} \begin{array}{ll} \Vert \mathbf {e}_1\Vert ^2_{L^2(\mathcal {V} )\otimes (S)_{-1}} &{}= \sum \limits _{k\in \mathbb N} \left\| \mathbf {e}_{1, \varepsilon ^{(k)}} \right\| ^2_{L^2(\mathcal {V} )} (2k)^{-p} + \sum \limits _{|\alpha |\not =1} \left\| \mathbf {e}_{1, \alpha } \right\| ^2_{L^2(\mathcal {V} )} (2\mathbb N)^{-p\alpha } \\ &{} = \sum \limits _{k\in \mathbb N} \left\| \mathbf {e}_{1, \varepsilon ^{(k)}} \right\| ^2_{L^2(\mathcal {V} )} (2k)^{-p} \end{array} \end{aligned}$$

and similarly

$$\begin{aligned} \left\| \mathbf {e}_1\right\| ^2_{C(T;\mathcal H)\otimes (S)_{-1}} = \sum _{k\in \mathbb N} \left\| \mathbf {e}_{1, \varepsilon ^{(k)}} \right\| ^2_{C(T;\mathcal H)} (2k)^{-p} \end{aligned}$$

also holds, then by (43) we obtain

$$\begin{aligned}&\Vert \mathbf {e}_1\Vert ^2_{C(T;\mathcal H) \otimes (S)_{-1, -p}} + \Vert \mathbf {e}_1\Vert ^2_{L^2(\mathcal {V} ) \otimes (S)_{-1, -p}} \\&\quad = \sum \limits _{k=1}^\infty \left\| \mathbf {e}_{1, \varepsilon ^{(k)}} \right\| ^2_{C(T;\mathcal H)} (2k)^{-p} + \sum \limits _{k=1}^\infty \left\| \mathbf {e}_{1, \varepsilon ^{(k)}} \right\| ^2_{L^2(\mathcal {V} )} (2k)^{-p} \\&\quad = \sum \limits _{k=1}^\infty \left( \left\| \mathbf {e}_{1, \varepsilon ^{(k)}} \right\| ^2_{C(T;\mathcal H)} + \left\| \mathbf {e}_{1, \varepsilon ^{(k)}} \right\| ^2_{L^2(\mathcal {V} )} \right) \, (2k)^{-p} \\&\quad \lesssim \sum \limits _{k\in \mathbb N}\, \left( \left\| m^{(1)}_k \right\| _{L^2(\mathcal {Q} ^*)} + \left\| m^{(2)}_k \right\| _{L^2(\mathcal {Q} ^*)} \right) \, (2k)^{-p} < \infty \end{aligned}$$

and the estimate (41) follows. $\square$

### Convergence of the truncated expansion

In practice, only the coefficients $u_\alpha$, $\lambda _\alpha$ for multi-indices of a maximal length *P*, i.e., up to a certain order *P*, can be computed. Thus, the infinite sum has to be truncated such that a given tolerance is achieved. Clearly, denoting by $\tilde{u}$ the approximated (truncated) solution and $u_r$ the truncation error, i.e.,

$$\begin{aligned} \tilde{u} = \sum _{|\alpha |\le P} \, u_\alpha \otimes H_\alpha \qquad \text {and} \qquad u_r = \sum _{|\alpha | > P} \, u_\alpha \otimes H_\alpha , \end{aligned}$$

we can represent the process as $u= \tilde{u} + u_r$. In applications, one computes $u_\alpha$ for $|\alpha |<P$ such that the desired bound $\Vert u_r\Vert _{\mathcal {V} \otimes L^2(\Omega )} =\Vert u-\tilde{u}\Vert _{\mathcal {V} \otimes L^2(\Omega ) } \le \epsilon$ is carried out. Convergence in $L^2$ is attained if the sum is truncated properly [24, 33, 46]. The truncation procedure relies on the regularity of the solution, the type of noise, and the discretization method for solving the deterministic equations involved, see e.g. [8] for finite element methods. Numerical treatment of elliptic PDEs perturbed by Gaussian noise with error estimate in appropriate weighted space of stochastic processes is presented in [45].
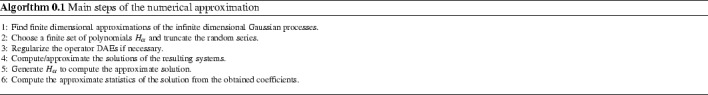


Similar results for specific equations can be found, e.g., in [1, 7, 9]. A general truncation method is stated in [24]. The same ideas can be applied to our equations once we have performed the regularization to the deterministic system (such that operator DAE is well-posed in each level), the convergence of the truncated expansion is, in general, guaranteed by the stability result of Theorem 6. The main steps of the numerical approach are sketched in Algorithm 3.1.

## More general cases

This section is devoted to the discussion of two generalizations. First, we consider general coordinatewise operators instead of simple coordinatewise operators as in the previous section. Thus, following the definition from Sect. 3.1.4, we allow the operators $\mathcal {K}$ and $\mathcal {B}$ to be composed out from families of deterministic operators $\{K_\alpha \}_{\alpha \in \mathcal I}$ and $\{B_\alpha \}_{\alpha \in \mathcal I}$, respectively, which may not be the same for all multi-indices. Second, we replace the Gaussian noise term by a stochastic integral term. The mean dynamics will remain unchanged, while the perturbation in the differential equation will be given in the form of a stochastic convolution.

Throughout this section we keep the following assumptions.

### Assumption 3

1. The operator $\mathcal K$ is a coordinatewise operator that corresponds to a family $\{K_\alpha \}_{\alpha \in \mathcal I}$ of deterministic operators $K_\alpha :\mathcal {V} \rightarrow \mathcal {V} ^*$, $\alpha \in \mathcal I$. The operators $K_\alpha$, $\alpha \in \mathcal I$, are linear, continuous, and positive on the kernel of *B*.
2. The constraint operator $\mathcal B$ is a coordinatewise operator that corresponds to a family $\{B_\alpha \}_{\alpha \in \mathcal I}$ of deterministic operators $B_\alpha :\mathcal {V} \rightarrow \mathcal {Q} ^*$, $\alpha \in \mathcal I$. The opertors $B_\alpha$ are linear and for every $\alpha \in \mathcal I$ there exists a right-inverse which is denoted by $B_\alpha ^-$.
3. The operators $K_\alpha$ and $B_\alpha$ are uniformly bounded.
4. The stochastic processes $\mathcal {F}$ and $\mathcal {G}$ are given in their chaos expansion forms (16) such the conditions (17) hold.

### Coordinatewise operators

In the given application, we consider the coordinatewise operators $\mathcal K$, $\mathcal B$ with

$$\begin{aligned} \mathcal {K} u=\sum _{\alpha \in \mathcal I}K_\alpha u_\alpha \otimes H_\alpha \qquad \text {and} \qquad \mathcal {B} u =\sum _{\alpha \in \mathcal I} B_\alpha u_\alpha \otimes H_\alpha \end{aligned}$$

such that Assumption 3 holds and the processes $G_t^{(1)}$ and $G_t^{(2)}$ are of the forms (31) and (36). This also implies that $\mathcal B^*$ is a coordinatewise operator, which corresponds to the family of operators $\{B_\alpha ^*\}_{\alpha \in \mathcal I}$ such that for $\lambda = \sum _{\alpha \in \mathcal I} \, \lambda _\alpha H_\alpha$ it holds that

$$\begin{aligned} \mathcal {B}^* \lambda =\sum _{\alpha \in \mathcal I} B^*_\alpha \lambda _\alpha \otimes H_\alpha . \end{aligned}$$

The chaos expansion method applied to the system with the Gaussian noise in the constraint equation (30) then leads to the following deterministic systems:

1. for $$|\alpha | =0$$, i.e., for $$\alpha = \mathbf {0}$$,
  $$\begin{aligned} \dot{u}_{\mathbf {0} }(t) + {K}_\mathbf {0} u_{\mathbf {0} }(t) + {B}_\mathbf {0}^* \lambda _{\mathbf {0} } (t)&= f_{\mathbf {0}}(t),\\ {B}_\mathbf {0} u_{\mathbf {0} }(t)&= g_{\mathbf {0}}(t), u_{\mathbf {0}} = u^0_{\mathbf {0}}. \end{aligned}$$
2. for $$|\alpha | =1$$, i.e., for $$\alpha = \varepsilon ^{(k)}$$, $$k\in \mathbb N$$,
  $$\begin{aligned} \dot{u}_{\varepsilon ^{(k)}}(t) + {K}_{\varepsilon ^{(k)}} u_{\varepsilon ^{(k)} }(t) + {B}^*_{\varepsilon ^{(k)}} \lambda _{\varepsilon ^{(k)} } (t)&= f_{\varepsilon ^{(k)}}(t) + m_k^{(1)}(t),\\ {B}_{\varepsilon ^{(k)}} u_{\varepsilon ^{(k)}}(t)&= g_{\varepsilon ^{(k)}}(t) + m_k^{(2)}(t), \end{aligned}$$
  with $$u_{\varepsilon ^{(k)} } (0) = u^0_{\varepsilon ^{(k)}}.$$
3. for the remaining $|\alpha |>1$,
  $$\begin{aligned} \dot{u}_{\alpha }(t) + {K}_\alpha u_{\alpha }(t) + {B}_\alpha ^* \lambda _{\alpha } (t)&= f_\alpha (t), \\ {B}_\alpha u_{\alpha }(t)&= g_{\alpha }(t), u_{\alpha }(0) = u_{\alpha }^0. \end{aligned}$$

As before, these systems may be solved in parallel. Furthermore, since the constraint equation includes again a perturbation, a regularization as in Sect. 3.5 is necessary. We omit further details here.

### Stochastic convolution

Consider the problem (7, where the stochastic disturbance is given in terms of a stochastic convolution term. More precisely, we are dealing with the problem of the form

44 $$\begin{aligned} \dot{u}(t) + \mathcal {K} u(t) + \mathcal {B}^* \lambda (t)&= \mathcal {F}(t)+ \delta (\mathcal {C} u), \nonumber \\ \mathcal {B} u(t)&= \mathcal {G}(t) + G^{(1)}_t \end{aligned}$$

with a consistent initial condition $u(0)=u^0$. We assume that Assumption 3 holds for operators $\mathcal K$ and $\mathcal B$ and processes $\mathcal F$ and $\mathcal G$. Additionally, we assume that $G_t^{(1)}$ is a Gaussian noise as in (12). The term $\delta (\mathcal C u)$ stays for an *Itô-Skorokhod stochastic integral*. The Skorokhod integral is a generalization of the Itô integral for processes which are not necessarily adapted. The fundamental theorem of stochastic calculus connects the Itô-Skorokhod integral with the Wick product by

45 $$\begin{aligned} \delta (\mathcal C u) =\int _{\mathbb R} \mathcal Cu \, \text {d}B_t = \int _{\mathbb R} \mathcal Cu \lozenge W_t \ \text {d}t , \end{aligned}$$

where the integral on the right-hand side of the relation is the Riemann integral and the derivative is taken in sense of distributions [22]. We assume that the operator $\mathcal C$ is a linear coordinatewise operator composed of a family of uniformly bounded operators $\{C_\alpha \}_{\alpha \in \mathcal I}$ such that $\mathcal C u$ is integrable in the Skorokhod sense [22]. The stochastic integral is the Itô-Skorokhod integral and it exists not only for processes adapted to the filtration but also for non-adapted ones. It is equal to the Riemann integral of a process $\mathcal C u$, stochastically convoluted with a singular white noise.

The operator $\delta$ is the adjoint operator of the Malliavin derivative $\mathbb D$. Their composition is known as the *Ornstein-Uhlenbeck operator* $\mathcal R$ which is a self-adjoint operator. These operators are the main operators of an infinite dimensional stochastic calculus of variations called the *Malliavin calculus* [37]. We consider these operators in Sect. 5.

For adapted processes *v* the Itô integral and the Skorokhod integral coincide, i.e., $I(v) = \delta (v)$. Because of this fact, we refer to the stochastic integral as the Itô-Skorokhod integral. Applying the definition of the Wick product (14) to the chaos expansion representation (9) of a process *v* and the representation (11) of a singular white noise in the definition (45) of $\delta (v)$, we obtain a chaos expansion representation of the Skorokhod integral. Clearly, for $v=\sum _{\alpha \in \mathcal I} v_\alpha (t) H_\alpha$ we have

$$\begin{aligned} v \, \lozenge {W}_t&= \sum \limits _{\alpha \in \mathcal I} \sum \limits _{k\in \mathbb {N}} v_\alpha (t) \xi _k(t) \, H_{\alpha + \varepsilon ^{(k)}} (\omega ) , \end{aligned}$$

and thus, it holds that

46 $$\begin{aligned} \delta (v)&= \sum \limits _{\alpha \in \mathcal I} \, \sum \limits _{k\in \mathbb {N}} \, v_{\alpha , k}\, H_{\alpha + \varepsilon ^{(k)}} (\omega ). \end{aligned}$$

Therein, we have used that $v_\alpha (t) = \sum _{k\in \mathbb N} v_{\alpha , k} \, \xi _k(t) \in L^2(\mathbb R)$ is the chaos expansion representation of $v_\alpha$ in the orthonormal Hermite functions basis with coefficients $v_{\alpha , k}\in \mathbb R$. Therefore, we are able to represent stochastic perturbations appearing in the stochastic equation (44) explicitly. Note that $\delta (v)$ belongs to the Wiener chaos space of higher order than *v*, see also [22, 28].

#### Definition 2

We say that a $L^2(\mathbb R)$-valued stochastic process $v =\sum _{\alpha \in \mathcal {I}} v_\alpha \, H_\alpha$, with coefficients $v_\alpha (t) = \sum _{k\in \mathbb {N}} v_{\alpha , k}\, \xi _k(t)$, $v_{\alpha , k}\in \mathbb R$, for all $\alpha \in \mathcal {I}$ is integrable in the Itô-Skorokhod sense if it holds that

47 $$\begin{aligned} \sum _{\alpha \in \mathcal {I}}\sum _{k\in \mathbb {N}} \, v^2_{\alpha , k} \,\, |\alpha | \,\, \alpha !\, < \, \infty . \end{aligned}$$

Then, the Itô-Skorokhod integral of *v* is of the form (46) and we write $v\in {{\mathrm{Dom}}}(\delta )$.

#### Theorem 10

The Skorokhod integral $\delta$ of an $L^2(\mathbb R)$-valued stochastic process is a linear and continuous mapping

$$\begin{aligned} \delta :{{\mathrm{Dom}}}(\delta ) \, \, \rightarrow \, \, L^2(\Omega ). \end{aligned}$$

#### Proof

Let *v* satisfy condition (47). Then we have

$$\begin{aligned} \Vert \delta (v) \Vert ^2_{L^2(\Omega )}&= \Big \Vert \sum \limits _{|\beta |>0} \, \sum \limits _{k \in \mathbb {N}} v_{\beta -\varepsilon ^{(k)}, k} H_\beta \Big \Vert ^2_{L^2(\Omega )} = \sum \limits _{|\beta |>0} \, \left( \sum \limits _{k \in \mathbb {N}} v_{\beta -\varepsilon ^{(k)}, k} \right) ^2 \, \beta ! \\&= \sum \limits _{\alpha \in \mathcal {I}} \,\left( \sum \limits _{k \in \mathbb {N}} v_{\alpha , k} \sqrt{\alpha _k+ 1}\right) ^2 \, \alpha ! \le c\, \sum \limits _{\alpha \in \mathcal {I}} \, \sum \limits _{k \in \mathbb {N}} v^{2}_{\alpha , k} \, |\alpha | \, \, \alpha ! < \infty , \end{aligned}$$

where we used $\beta ! = (\alpha + \varepsilon ^{(k)})! = (\alpha _k + 1) \, \alpha !$, for $\alpha \in \mathcal I$, $k\in \mathbb N$. $\square$

A detailed analysis of the domain and the range of operators of the Malliavin calculus in spaces of stochastic distributions can be found in [28, 29, 31].

First, we solve the stochastic operator DAE (44) with the stochastic perturbations given in terms of a stochastic convolution and without disturbance in the constraint equation. In order to prove the convergence of obtained solution in the Kondratiev space of generalized processes it is necessary to assume uniform boundness of the family of operators $C_{\alpha }$, $\alpha \in \mathcal I$. Then, we consider the stochastic operator DAE (44) with perturbation in the constraint equation that is given by a Gaussian noise term.

#### Theorem 11

Let Assumption 3 hold for the operators $\mathcal {K}$ and $\mathcal {B}$ and stochastic processes $\mathcal {F}$ and $\mathcal {G}$ and let $\mathcal C$ be a coordinatewise operator that corresponds to a family of deterministic operators $\{C_\alpha \}_{\alpha \in \mathcal I}$, $C_\alpha : \mathcal {V} \rightarrow \mathcal {V} ^*$ for $\alpha \in \mathcal I$ that satisfy

48 $$\begin{aligned} \Vert C_\alpha \Vert \le d < 1, \qquad \text {for all} \, \, \, \, \alpha \in \mathcal I . \end{aligned}$$

Then, for any consistent initial data that satisfies (21) there exists a unique solution $u\in L^2(T;\mathcal {V} )\otimes (S)_{-1}$ of the stochastic operator DAE

49a $$\begin{aligned} \dot{u}(t)\ + \mathcal {K} u(t)\ +\ \mathcal {B} ^* \lambda (t)&=\mathcal {F} (t) + \delta (\mathcal {C} u), \end{aligned}$$

49b $$\begin{aligned} \mathcal {B} u(t)&= \mathcal {G} (t) . \end{aligned}$$

#### Proof

We are looking for the solution in the chaos expansion form (9). For this, we apply the polynomial chaos expansion method to problem (49 and obtain the following systems of deterministic operator DAEs:

- $$1^\circ $$ for $$|\alpha | =0$$, i.e., for $$\alpha = \mathbf {0}$$,
  50 $$\begin{aligned} \dot{u}_{\mathbf {0} }(t) + {K}_{\mathbf {0} } u_{\mathbf {0} }(t) +{B}_{\mathbf {0} }^* \lambda _{\mathbf {0} } (t)= & {} f_\mathbf {0}(t),\nonumber \\ {B}_{\mathbf {0} } u_{\mathbf {0} }(t)= & {} g_\mathbf {0}(t). \end{aligned}$$
- $$2^\circ $$ for $$|\alpha | =1$$, i.e., for $$\alpha = \varepsilon ^{(k)}$$, $$k\in \mathbb N$$,
  51 $$\begin{aligned} \begin{array}{rrl} \dot{u}_{\varepsilon ^{(k)} }(t) + {K}_{\varepsilon ^{(k)} }\, u_{\varepsilon ^{(k)} }(t) +{B}_{\varepsilon ^{(k)} }^* \, \lambda _{\varepsilon ^{(k)} } (t) &{} = &{} f_{\varepsilon ^{(k)}} + (C_{\mathbf {0}} \, u_{\mathbf {0}})_k, \\ {B}_{\varepsilon ^{(k)} } \, u_{\varepsilon ^{(k)}}(t) &{} = &{} g_{\varepsilon ^{(k)}}(t) . \end{array} \end{aligned}$$
- $$3^\circ $$ for $$|\alpha |>1$$,
  52 $$\begin{aligned} \dot{u}_{\alpha }(t) + {K}_{\alpha } \, u_{\alpha }(t) + {B}_{\alpha }^* \, \lambda _{\alpha } (t)= & {} f_\alpha (t) + \sum \limits _{k\in \mathbb N} \, \left( C_{\alpha - \varepsilon ^{(k)} } \, u_{\alpha -\varepsilon ^{(k)}}\right) _k, \nonumber \\ {B}_{\alpha } \, u_{\alpha }(t)= & {} g_{\alpha }(t) . \end{aligned}$$

Note that the corresponding initial conditions are given as in systems (32)–(34). The term $(C_{\mathbf {0}}u_{\mathbf {0}})_k$ appearing in (51) represents the *k*th component of the action of the operator $C_{\mathbf {0}}$ on the solution $u_{\mathbf {0}}$ obtained in the previous step, i.e., on the solution of the system (50). Similarly, the term $(C_{\alpha -\varepsilon ^{(k)} } \, u_{\alpha -\varepsilon ^{(k)}})_k$ from (52) represents the *k*th coefficient obtained by the action of the operator $C_{\alpha - \varepsilon ^{(k)} }$ on $u_{\alpha - \varepsilon ^{(k)} }$ calculated in the previous steps. We use the convention that $C_{\alpha - \varepsilon ^{(k)} }$ exists only for those $\alpha \in \mathcal I$ for which $\alpha _k \ge 1$. Therefore, the sum $\sum _{k\in \mathbb N} (C_{\alpha - \varepsilon ^{(k)} } \, u_{\alpha -\varepsilon ^{(k)}})_k$ has as many summands as the multi-index $\alpha$ has non-zero components. For example, for $\alpha =(2, 0, 1, 0, 0,\ldots )$ with two non-zero components $\alpha _1=2$ and $\alpha _3=1$, the sum has two terms $(C_{(1, 0, 1, 0, 0,\ldots )} u_{(1, 0, 1, 0, 0,\ldots )})_1$ and $(C_{(2, 0, 0, 0, 0,\ldots )} u_{(2, 0, 0, 0, 0,\ldots )})_3$.

We point out that, in contrast to the previous cases, the unknown coefficients are obtained by recursion. Thus, in order to calculate $u_\alpha$, we need the solutions $u_\beta$ for $\beta <\alpha$ from the previous steps. Also this case can be found in applications, see for example [24, 29, 31, 33].

We apply the estimate (2) from Theorem 1 to the deterministic operator DAEs (50)–(52) for the coefficients $u_\alpha$ in each step recursively and then prove the convergence of *u* in $L^2(T;\mathcal {V} )\otimes (S)_{-1}$. Particularly, we have to show that for some $p\in \mathbb N$ it holds

$$\begin{aligned} \sum _{\alpha \in \mathcal I} \Vert u_\alpha \Vert ^2_{L^2(\mathcal {V} )} (2\mathbb N)^{- p \alpha } < \infty . \end{aligned}$$

For $|\alpha |=0$, from the system (50) and by (2) we estimate the coefficient $u_{\mathbf {0}}$, i.e.,

$$\begin{aligned} \Vert u_{\mathbf {0}} \Vert _{L^2(\mathcal {V} )}^2 \lesssim \Vert u^0_{B,\mathbf {0}} \Vert _\mathcal {H} ^2 + \Vert f_\mathbf {0}\Vert ^2_{L^2(\mathcal {V} ^*)} + \Vert g_\mathbf {0}\Vert ^2_{H^1(\mathcal {Q} ^*)}. \end{aligned}$$

For $|\alpha |=1$, i.e., for $\alpha =\varepsilon ^{(k)}$, $k\in \mathbb N$, by the system (51) we obtain the estimate

$$\begin{aligned} \Vert u_{\varepsilon ^{(k)}} \Vert _{L^2(\mathcal {V} )}^2 \lesssim \Vert u^0_{B,\varepsilon ^{(k)}}\Vert _\mathcal {H} ^2 + \Vert f_{\varepsilon ^{(k)}} + (C_{\mathbf {0}} u_{\mathbf {0}})_k \Vert ^2_{L^2(\mathcal {V} ^*)} + \Vert g_{\varepsilon ^{(k)}}\Vert ^2_{H^1(\mathcal {Q} ^*)},\ \ k\in \mathbb N, \end{aligned}$$

while for $|\alpha |>1$ from (52) we obtain

$$\begin{aligned} \Vert u_{\alpha } \Vert _{L^2(\mathcal {V} )}^2 \lesssim \Vert u^0_{B,\alpha }\Vert _\mathcal {H} ^2 + \big \Vert f_\alpha + \sum _{k\in \mathbb N} \, \, (C_{\alpha -\varepsilon ^{(k)}} u_{\alpha -\varepsilon ^{(k)}} )_k \big \Vert ^2_{L^2(\mathcal {V} ^*)} + \Vert g_\alpha \Vert ^2_{H^1(\mathcal {Q} ^*)}. \end{aligned}$$

We sum up all the coefficients and apply the obtained estimates. Thus, we get

53 $$\begin{aligned} \sum \limits _{\alpha \in \mathcal I} \Vert u_\alpha \Vert ^2_{L^2(\mathcal {V} )} (2\mathbb N)^{- p\alpha }\lesssim & {} \sum \limits _{\alpha \in \mathcal I} \Vert u^0_{B,\alpha } \Vert _\mathcal {H} ^2 (2\mathbb N)^{-p\alpha } + \sum \limits _{\alpha \in \mathcal I} \Vert f_\alpha \Vert ^2_{L^2(\mathcal {V} ^*)} (2\mathbb N)^{-p\alpha } \nonumber \\&+ \sum \limits _{\alpha \in \mathcal I} \Vert g_\alpha \Vert ^2_{H^1(\mathcal {Q} ^*)} (2\mathbb N)^{-p\alpha } \nonumber \\&+ \sum \limits _{\alpha \in \mathcal I, |\alpha |>0} \, \left( \sum \limits _{k\in \mathbb N} \, (C_{\alpha -\varepsilon ^{(k)}} u_{\alpha -\varepsilon ^{(k)}})_k \right) ^2 \, (2\mathbb N)^{-p\alpha }.\quad \end{aligned}$$

From the assumptions (17) and (22) it follows that the first three summands on the right hand side of (53) are finite. The last term can be estimated in the following way

$$\begin{aligned} \begin{array}{ll} &{}\sum \limits _{|\alpha |>0} \left( \sum \limits _{k\in \mathbb N} \left( C_{\alpha -\varepsilon ^{(k)}}u_{\alpha -\varepsilon ^{(k)}}\right) _k \right) ^2 (2\mathbb N)^{-p\alpha } \le \sum \limits _{\beta \in \mathcal I}\left( \sum \limits _{k\in \mathbb N} \left( C_{\beta }u_{\beta }\right) _k \, (2k)^{-\frac{p}{2}}\right) ^2(2\mathbb N)^{-p\beta }\\ &{} \qquad \le \sum \limits _{\beta \in \mathcal I} \left( \sum \limits _{k\in \mathbb N} \left( C_{\beta } u_{\beta }\right) _k^2 \sum \limits _{k\in \mathbb N} (2k)^{-p}\right) (2\mathbb N)^{-p \beta } \le M\, \sum \limits _{\beta \in \mathcal I} \, \, \Vert C_{\beta } u_{\beta }\Vert ^2 \, (2\mathbb N)^{-p\beta } \\ &{}\qquad \le M\, d\, \sum \limits _{\beta \in \mathcal I} \, \, \Vert u_{\beta }\Vert ^2 \, (2\mathbb N)^{-p\beta } = M\, d\, \Vert u\Vert ^2_{L^2{(\mathcal {V} )} \otimes (S)_{-1, -p}}. \end{array} \end{aligned}$$

Therein, we have first used the substitution $\alpha =\beta + \varepsilon ^{(k)}$ and the property

$$\begin{aligned} (2\mathbb N)^{\beta +\varepsilon ^{(k)}} = (2\mathbb N)^{\beta } \cdot (2\mathbb N)^{\varepsilon ^{(k)}} =(2\mathbb N)^{\beta } \cdot (2k), \end{aligned}$$

then the Cauchy-Schwartz inequality, the uniformly boundness of the family $\{C_\alpha \}_{\alpha \in \mathcal I}$ from (48), and at last the sum $M= \sum _{k\in \mathbb N}\, (2k)^{-p} < \infty$ for $p>1$. Finally, putting everything together in (53), we obtain

$$\begin{aligned} \begin{array}{ll} &{}\Vert u\Vert ^2_{L^2(\mathcal {V} )\otimes (S)_{-1}} \le c \, \left( \, \sum \limits _{\alpha \in \mathcal I} \left\| u^0_{B,\alpha } \right\| _\mathcal {H} ^2 (2\mathbb N)^{-p\alpha } + \sum \limits _{\alpha \in \mathcal I} \left\| f_\alpha \right\| ^2_{L^2(\mathcal {V} ^*)} (2\mathbb N)^{-p\alpha } \right. \\ &{}\left. \qquad \qquad \qquad \qquad \quad + \sum \limits _{\alpha \in \mathcal I} \Vert g_\alpha \Vert ^2_{H^1(\mathcal {Q} ^*)} (2\mathbb N)^{-p\alpha } \right) + M\, d\, \Vert u\Vert ^2_{L^2(\mathcal {V} )\otimes (S)_{-1}} . \end{array} \end{aligned}$$

We group the two summands with the term $\Vert u\Vert ^2_{L^2(\mathcal {V} )\otimes (S)_{-1}}$ on the left hand side of the inequality and obtain

$$\begin{aligned} \begin{array}{ll} \Vert u\Vert ^2_{L^2(\mathcal {V} )\otimes (S)_{-1}} (1-Md) &{}\lesssim \sum \limits _{\alpha \in \mathcal I} \left\| u^0_{B,\alpha } \right\| _\mathcal {H} ^2 (2\mathbb N)^{-p\alpha } + \sum \limits _{\alpha \in \mathcal I} \Vert f_\alpha \Vert ^2_{L^2(\mathcal {V} ^*)} (2\mathbb N)^{-p\alpha } \\ &{}\quad \ +\sum \limits _{k=1}^\infty \Vert m_k \Vert ^2_{L^2(\mathcal {V} ^*)} (2k)^{-p} + \sum \limits _{\alpha \in \mathcal I} \Vert g_\alpha \Vert ^2_{H^1(\mathcal {Q} ^*)} (2\mathbb N)^{-p\alpha }. \end{array} \end{aligned}$$

Since (48) holds, one can choose *p* large enough so that $1 - Md >0$. With this, we have proven that the solution *u* of (49 the norm $\Vert u\Vert ^2_{{L^2(\mathcal {V} )\otimes (S)_{-1}}}$ is finite and thus, complete the proof of theorem norm. $\square$

Let us now consider briefly the stochastic operator DAE (44). This problem corresponds to the stochastic operator DAE (49 with additional disturbance in the constraint equation. Similar to Theorem 9, the regularization is needed and will be provided only for the coefficients $u_\alpha$, when $|\alpha |=1$. Thus one can obtain the error estimate of the solution of the initial problem (49 and the perturbed one (44), i.e. of the solutions of their corresponding problems in extended forms. Here we state the theorem, but omit the proof.

#### Theorem 12

Let the assumptions of Theorem 11 hold. Let $({u}_1, {u}_2, {v}_2, {\lambda }_2)$ be the solution of operator DAE

$$\begin{aligned} {{\dot{u}}_{1}}(t) + v_{2}(t) + {\mathcal {K} {\big (u_1(t)+u_2(t)\big )}} + {\mathcal {B} }^{*} {\lambda (t)}&= {\mathcal {F} (t)} + {\delta (C u)} \\ {\mathcal {B} } u_2(t)&= {\mathcal {G} (t)} \\ {\mathcal {B} } v_2(t)&= {\dot{\mathcal {G} }}(t) \end{aligned}$$

and $({\hat{u}}_{1}, {\hat{u}}_{2}, {\hat{v}}_{2}, {\hat{\lambda }}_{2})$ the solution of the corresponding perturbed operator DAE

$$\begin{aligned} {{\dot{u}}_{1}(t)} + v_{2}(t) + {\mathcal {K} {\big (u_1(t)+u_2(t)\big )}} + {{\mathcal {B} }^{*}} {\lambda } (t)&= {\mathcal {F} (t)} + {\delta (C u)} \\ {\mathcal {B} } u_{2}(t)&= {\mathcal {G} (t)} + G_{t}^{(1)} \\ {\mathcal {B} } v_{2}(t)&= {\dot{\mathcal {G} }}(t) + G_{t}^{(1)}, \end{aligned}$$

where the perturbations $G_{t}^{(1)}$ and $G_{t}^{(2)}$ are considered to be of the forms (31) and (36), with the coefficients $m^{(1)}_k \in L^2(T;{\mathcal {Q} }^{*})$ and $m^{(2)}_k \in L^2(T;{\mathcal {Q} }^{*})$ such that (40) holds for some $p\in \mathbb N_0$. . Then, the error $\mathbf {e}_1 = {\hat{u}}_1 - u_1$ satisfies the following estimate

$$\begin{aligned} \begin{array}{ll} &{}\Vert {\mathbf {e}}_1\Vert ^2_{C(T;\mathcal H) \otimes (S)_{-1, -p}} + \Vert {\mathbf {e}}_1\Vert ^2_{L^2(\mathcal {V} ) \otimes (S)_{-1, -p}} \\ &{}\qquad \qquad \lesssim \sum \limits _{k\in \mathbb N}\, \left\| m^{(1)}_k \right\| _{L^2(\mathcal {Q} ^*)} (2k)^{-p} + \sum \limits _{k\in \mathbb N}\, \left\| m^{(2)}_k \right\| _{L^2(\mathcal {Q} ^*)} (2k)^{-p} < \infty . \end{array} \end{aligned}$$

With this result, we close this section and consider a further generalization, namely the fully stochastic case.

## An example involving operators of Malliavin calculus

We present an example involving operators of Malliavin calculus which has the same structure as the deterministic operator DAE (1. Although this example does not arise in fluid dynamics it is related with the extension of our results to nonlinear equations in particular Navier–Stokes equation. Thus, we consider a semi-explicit systems including the stochastic operators from the Malliavin calculus and use their duality relations. Denote by $\mathbb D$ and $\delta$ the Malliavin derivative operator and the Itô-Skorokhod integral, respectively. As mentioned above, the Itô-Skorokhod integral is the adjoint operator of the Malliavin derivative, i.e., the duality relationship

$$\begin{aligned} \mathbb E \left( F\cdot \delta (u)\right) \, = \, \mathbb E \left( \langle \mathbb D F, \, u\rangle \right) , \end{aligned}$$

holds for stochastic functions *u* and *F* belonging to appropriate spaces [37].

Assume that the stochastic operator $\mathcal K$ is a coordinatewise operator such that the corresponding deterministic operators $\{K_\alpha \}_{\alpha \in \mathcal I}$ are densely defined on a given Banach space *X*. Taking in (1 the operators $\mathcal B = \mathbb D$ and thus $\mathcal B^* = \delta$, we can consider the stochastic operator DAE of the form

54 $$\begin{aligned}&\displaystyle \dot{u} + \mathcal K \, u + \, \delta \, \lambda = v \nonumber \\&\displaystyle \mathbb D \, u = y \end{aligned}$$

such that the initial condition $u(0) = u^0$ holds and given stochastic processes *v* and *y*.

The results concerning the generalized Malliavin calculus and the equations involving these operators can be found in [28, 29, 31, 32]. The chaos expansion method combined with the regularization techniques presented in the previous sections can be applied also in this case. Here we present the direct chaos expansion approach and prove the convergence of the obtained solution.

In the generalized $S'(\mathbb R)$ setting, the operators of the Malliavin calculus are defined as follows:

1. The *Malliavin derivative*, namely $\mathbb D$, as a stochastic gradient in the direction of white noise, is a linear and continuous mapping $\mathbb {D}:{{\mathrm{Dom}}}(\mathbb D) \subseteq X\otimes (S)_{-1} \rightarrow X\otimes S'(\mathbb R) \otimes (S)_{-1}$ given by
  55 $$\begin{aligned} \mathbb {D} u=\sum _{\alpha \in \mathcal {I}}\sum _{k\in \mathbb {N}}\, \alpha _k \, u_\alpha \, \otimes \, \xi _k\, \otimes {H}_{\alpha -\varepsilon _k}, \end{aligned}$$
  for $u=\sum _{\alpha \in \mathcal I}u_\alpha \otimes H_\alpha$, $u_\alpha \in X$, $\alpha \in \mathcal I$. We say that a process *u* is differentiable in Malliavin sence, i.e., it belongs to the domain ${{\mathrm{Dom}}}(\mathbb D)$ if and only if for some $p\in \mathbb N_0$ it holds that
  $$\begin{aligned} \sum _{\alpha \in \mathcal I} \, |\alpha |^2 \, \Vert u_\alpha \Vert ^2_X \, (2\mathbb N)^{-p\alpha } < \infty . \end{aligned}$$
  The operator $\mathbb D$ reduces the order of the Wiener chaos space and it holds that the kernel ${{\mathrm{Ker}}}(\mathbb D)$ consists of constant random variables, i.e., random variables having the chaos expansion in the Wiener chaos space of order zero. In terms of quantum theory, this operator corresponds to the annihilation operator.
2. The *Itô-Skorokhod integral*, namely $\delta$, is a linear and continuous mapping $\delta :X\otimes S'(\mathbb R) \otimes (S)_{-1}\rightarrow X\otimes (S)_{-1}$ given by
  $$\begin{aligned} \delta (F )=\sum _{\alpha \in \mathcal {I}}\sum _{k\in \mathbb {N}} f_{\alpha }\otimes v_{\alpha ,k} \otimes H_{\alpha +\varepsilon _k}, \,\,\text{ for } F=\sum _{\alpha \in \mathcal I} f_\alpha \otimes \left( \sum _{k\in \mathbb {N}}v_{\alpha , k}\, \xi _k\right) \otimes H_\alpha . \end{aligned}$$
  Note that the domain ${{\mathrm{Dom}}}(\delta ) = X\otimes S'(\mathbb R) \otimes (S)_{-1}$. The operator $\delta$ is the adjoint operator of the Malliavin derivative. It increases the order of the Wiener chaos space and in terms of quantum theory $\delta$ corresponds to the creation operator.
3. The *Ornstein-Uhlenbeck operator*, namely $\mathcal R$, as the composition $\delta \circ \mathbb D$, is the stochastic analogue of the Laplacian. It is a linear and continuous mapping $\mathcal R:X\otimes (S)_{-1}\rightarrow X\otimes (S)_{-1}$ given by
  $$\begin{aligned} \mathcal R(u) = \sum _{\alpha \in \mathcal I}|\alpha |u_\alpha \otimes H_\alpha \quad \text{ for } u=\sum _{\alpha \in \mathcal I}u_\alpha \otimes H_\alpha . \end{aligned}$$
  Clearly, $\mathcal R$ is a coordinatewise operator and its domain ${{\mathrm{Dom}}}(\mathcal R)$ coincides with the domain ${{\mathrm{Dom}}}(\mathbb D)$. In terms of quantum theory, the operator $\mathcal R$ corresponds to the number operator. It is a self-adjoint operator with eigenvectors equal to the basis elements $H_\alpha$, $\alpha \in \mathcal I$, i.e., $\mathcal R(H_\alpha )=|\alpha |H_\alpha$, $\alpha \in \mathcal I$. Therefore, Gaussian processes from the Wiener chaos space of order one with zero expectation are the only fixed points for the Ornstein-Uhlenbeck operator [28, 31].

In this section we present the direct method of solving system (54), which relies on the results obtained in [27, 29, 31]. First, we solve the second equation with the initial condition in (54) and obtain the solution *u* in the space of stochastic processes $X\otimes (S)_{-1}$. Then by subtracting the obtained solution *u* in the first equation of (54) we solve an integral equation and obtain the explicit form of $\lambda$ in the space of generalized $S'(\mathbb R)$-stochastic processes.

### Theorem 13

Let the operator $\mathcal K$ satisfy the assumptions $1^\circ$ and $3^\circ$ of Assumption 3. Let a process $y \in X \otimes S'(\mathbb R) \otimes (S)_{-1}$ have a chaos representation $y= \sum _{\alpha \in \mathcal I} \sum _{k\in \mathbb N} y_{\alpha , k} \otimes \xi _k \otimes H_{\alpha }$ and a process $v\in X\otimes (S)_{-1}$ have a chaos representation $v= \sum _{\alpha \in \mathcal I} v_{\alpha } \otimes H_{\alpha }$ such that $\mathbb E v = K_{\mathbf 0} \, u^0$. Then the stochastic problem (54) with the initial condition $\mathbb Eu= {u}^0\in X$ has a unique solution $u \in X\otimes (S)_{-1}$ and $\lambda \in X \otimes S (\mathbb {R}) \otimes (S)_{-1}$ given respectively by

56 $$\begin{aligned} u= \, {u}^{0} \, + \, \sum _{\alpha \in \mathcal {I}, |\alpha | > 0} \, \frac{1}{|\alpha |} \, \sum _{k\in \mathbb {N}} \, y_{\alpha - \varepsilon ^{(k)}, k} \, \, \otimes \, H_\alpha \end{aligned}$$

and

57 $$\begin{aligned} \lambda = \sum \limits _{\alpha \in \mathcal {I}} \, \sum \limits _{k\in \mathbb {N}} \, \, (\alpha _k+1) \, \, \frac{v^{(1)}_{\alpha + \varepsilon ^{(k)}}}{|\alpha + \varepsilon ^{(k)}|}\;\otimes \,\xi _k \, \otimes \, H_\alpha , \end{aligned}$$

where $$v^{(1)} = v - \dot{u} - \mathcal K u$$.

### Proof

We search for the solution represented of the form (18). The initial value problem involving the Malliavin derivative operator

58 $$\begin{aligned} \mathbb {D} u= y, \qquad \mathbb Eu= {u}^0 \in X \end{aligned}$$

can be solved by applying the integral operator on both sides of the equation. For a given process $y\in X\otimes {S}_{-l}(\mathbb {R}) \otimes (S)_{-1, -q}$, $l\in \mathbb N_0$, $q>l+1$, represented in its chaos expansion form $y=\sum _{\alpha \in \mathcal {I}}\sum _{k\in \mathbb {N}} y_{\alpha , k}\otimes \xi _k\otimes H_{\alpha }$, the equation (58) has a unique solution in ${{\mathrm{Dom}}}(\mathbb D)$ given by (56), [27, 29]. Clearly, it holds that

$$\begin{aligned} \Vert u\Vert ^2_{X\otimes (S)_{-1, -q}} \le \left\| u^0\right\| ^2_X \, + c \, \Vert y\Vert ^2_{X \otimes S_{-l}(\mathbb R) \otimes (S)_{-1, -q}} < \infty . \end{aligned}$$

The operator $\mathcal K$ is a coordinatewise operator and corresponds to a uniformly bounded family of operators $\{K_\alpha \}_{\alpha \in \mathcal I}$, i.e., it holds that $\Vert K_\alpha \Vert \le M$, $\alpha \in \mathcal I$. For $u\in X\otimes (S)_{-1} \bigcap {{\mathrm{Dom}}}(\mathbb D)$ it holds that

$$\begin{aligned} \Vert \mathcal K u\Vert ^2_{X\otimes (S)_{-1, -q}} = \sum _{\alpha \in \mathcal I} \left\| K_\alpha u_\alpha \right\| ^2_X \, (2\mathbb N)^{-q\alpha } \le M \Vert u\Vert ^2_{X\otimes (S)_{-1, -q}} < \infty \end{aligned}$$

and thus we conclude that $\mathcal K u \in X\otimes (S)_{-1, -q}$. Since $y_\alpha \in X\otimes S_{-l}(\mathbb R)$, we can apply the formula for derivatives of the Hermite functions [22]. Thus,

$$\begin{aligned} \dot{y}_\alpha = \sum _{k\in \mathbb N} \, y_{\alpha , k} \otimes \frac{d}{dt} \, \xi _k = \sum _{k\in \mathbb N} \, y_{\alpha , k} \, \otimes \left( \sqrt{\frac{k}{2}} \, \xi _{k-1} - \sqrt{\frac{k+1}{2}} \xi _{k+1} \right) \end{aligned}$$

and it holds that $\dot{y}_\alpha \in X\otimes S_{-l-1}(\mathbb R)$. We note that the problem $\mathbb D \dot{u} = \dot{y}$ with the initial condition $\mathbb E \dot{u} = {u^1}\in X$ also holds and it can be solved as equation (58). Hence, the following estimate holds

$$\begin{aligned} \Vert \dot{u}\Vert ^2_{X\otimes (S)_{-1, -q}} \le \left\| u^1\right\| ^2_X \, + c \, \Vert \dot{y}\Vert ^2_{X \otimes S_{-l-1}(\mathbb R) \otimes (S)_{-1, -q}} < \infty . \end{aligned}$$

Let $v\in X\otimes (S)_{-1, -q}$ and denote by $v^{(1)} = v- \dot{u} - \mathcal K u$. From the given assumptions it follows $v^{(1)} \in X\otimes (S)_{-1, -q}$ and it has zero expectation. Let

$$\begin{aligned} v_1 = \sum \limits _{\alpha \in \mathcal {I}, |\alpha | \ge 1} \, v^{(1)}_\alpha \,\otimes \, H_\alpha , \quad v^{(1)}_\alpha \in X. \end{aligned}$$

Then the integral equation

$$\begin{aligned} \delta \lambda \, = \, v_1 \end{aligned}$$

has a unique solution $\lambda$ in $X \otimes S_{-l-1}(\mathbb {R}) \otimes (S)_{-1, -q}$, for $l> q$, given in the form (57), see [28, 31]. The estimate

$$\begin{aligned} \Vert v\Vert ^2_{X\otimes (S)_{-1,-q}} \le c \, \left( \Vert u\Vert ^2_{X\otimes (S)_{-1,-q}} + \Vert v\Vert ^2_{X\otimes (S)_{-1,-q}} + \Vert \dot{u}\Vert ^2_{X\otimes (S)_{-1, -q}} \right) \end{aligned}$$

also holds. $$\square $$

### Theorem 14

Let $y= \sum _{\alpha \in \mathcal I} \sum _{k\in \mathbb N} y_{\alpha , k} \otimes \xi _k \otimes H_{\alpha }\in X \otimes S'(\mathbb R) \otimes (S)_{-1}$. The initial value problem (58) is equivalent to the system of two initial values problems

59 $$\begin{aligned} \mathbb D \, u_1 = 0 , \quad \mathbb E u_1 = u^0 \in X\qquad \text {and}\qquad \mathbb D \, u_2 = y , \quad \mathbb E u_2= 0, \end{aligned}$$

where $$u=u_1+u_2$$.

### Proof

Let $u_1$ and $u_2$ be the solutions of the system (59). From the linearity of the operator $\mathbb D$ and the linearity of $\mathbb E$ it follows $\mathbb D u = \mathbb D (u_1+u_2) = \mathbb D u_1 + \mathbb D u_2 = y$ and $\mathbb E u = \mathbb E (u_1+u_2) = \mathbb E u_1 + \mathbb E u_2 = u^0$. Thus the superposition of $u_1$ and $u_2$ solves (58).

Let now *u* be the solution of (58). By Theorem 13 it has chaos expansion representation form (56). The kernel of $\mathbb D$, i.e., ${{\mathrm{Ker}}}(\mathbb D)$ is equal to $\mathcal H_0$ and therefore *u* can be expressed in the form $u=u_1 + u_2$, where $u_1\in {{\mathrm{Ker}}}(\mathbb D)$ and $u_2\in {{\mathrm{Im}}}(\mathbb D)$. Thus, by (56) we conclude that $\mathbb D u_1 =0$ and $\mathbb E u_1 = u^0$, while $\mathbb D u_2 = y$ and $\mathbb E u_2 =0$. $\square$

### Extension to nonlinear equations

In [36] the authors show that a random polynomial nonlinearity can be expanded in a Taylor alike series involving Wick products and Malliavin derivatives. This result has been applied to the nonlinear advection term in the Navier–Stokes equations [44]. There a detailed study of the accuracy and computational efficiency of these Wick-type approximations is shown. We point out that following the same approach we can extend the ideas presented in this paper to Navier–Stokes equations. Specifically, by the product formula, of two square-integrable stochastic processes *u* and *v*,

$$\begin{aligned} uv = \sum _{i=0}^P \, \frac{\mathbb D^{(i)} u \, \lozenge \, \mathbb D^{(i)} v }{i!} , \end{aligned}$$

where $\lozenge$ denotes the Wick product and $\mathbb D^{(i)}$ is the *i*th order of the Malliavin derivative operator, one can construct approximations of finite stochastic order. Particularly, the nonlinear advection term in the Navier–Stokes equations can be approximated by

60 $$\begin{aligned} (u\cdot \nabla ) u \simeq \sum _{i=0}^Q \, \frac{ (\mathbb D^{(i)} \lozenge \nabla ) \, \mathbb D^{(i)} u }{i!}, \end{aligned}$$

where *Q* denotes the highest stochastic order in the Wick-Malliavin expansion. The zero-order approximation $(u\cdot \nabla ) u \simeq (u \lozenge \nabla ) u$ is known as the Wick approximation, while $(u\cdot \nabla ) u \simeq (u \lozenge \nabla ) u + (\mathbb D u \lozenge \nabla ) \, \mathbb D u$ is the first-order Wick-Malliavin approximation [44]. As the Malliavin derivate has an explicit chaos expansion representation form (55), the formula (60) allows us to express the nonlinear advection term in terms of chaos expansions. Therefore, the ideas presented in this paper for the linear semi-explicit stochastic operator DAEs can be extended to Navier–Stokes equations and in general to equations with nonlinearities of the type (60). Moreover, the multiplication formula

$$\begin{aligned} v \, G = v\lozenge G + \sum _{\alpha \in \mathcal I} \sum _{k\in \mathbb N} \, (\alpha _k + 1)\, v_{\alpha +\varepsilon ^{(k)}} \, g_k \, \, H_\alpha , \end{aligned}$$

holds for a Gaussian process $G = g_{\mathbf 0} + \sum _{k\in \mathbb N} \, g_k\, H_{\varepsilon ^{(k)}} \in X\otimes (S)_{-1}$ and a process $v =\sum _{\alpha \in \mathcal I} v_\alpha H_\alpha \in X\otimes (S)_{-1}$ [28, 31]. The equations involving higher orders of the Malliavin derivarive operator were solved in [31]. Thus, the results proved in this paper and the ones in [36, 44] can be generalized for this type of general processes (not necessary square integrable). We intent to investigate this in a future work.

## Conclusion

We have analyzed the influence of stochastic perturbations to linear operator DAEs of semi-explicit structure. With the application of the polynomial chaos expansion, we could reduce the problem to a system of deterministic operator DAEs. Since the obtained system is very sensitive to perturbations in the constraint equation, we analyze a regularized version of the system. With this, we have proven the existence and uniqueness of a solution of the stochastic operator DAE in a weighted space of generalized stochastic processes. Examples analyzed in this paper are the Stokes equations and the linearized Navier–Stokes equations. Moreover, the results of this paper can be extended to a certain type of nonlinear equations including Navier–Stokes.
